# Emergent ecology in a microscale model of the surface ocean

**DOI:** 10.1128/mbio.02372-24

**Published:** 2024-10-09

**Authors:** Falk Eigemann, Jutta Hoffmann, Charlotte Schampera, Shuting Liu, Luis M. Bolaños, Mats Heemeyer, Craig A. Carlson, Stephen Giovannoni, Ferdi L. Hellweger

**Affiliations:** 1 Water Quality Engineering, Technical University of Berlin, Berlin, Germany; 2 Marine Science Institute/Department of Ecology, Evolution and Marine Biology, University of California Santa Barbara, Santa Barbara, California, USA; 3 Department of Environmental & Sustainability Sciences, Kean University, Union, New Jersey, USA; 4 School of Biosciences, University of Exeter, Exeter, United Kingdom; 5 Department of Microbiology, Oregon State University, Corvallis, Oregon, USA; The University of Tennessee Knoxville, Knoxville, Tennessee, USA

**Keywords:** microbial ecology, biogeochemical model, agent-based model, attachment, chemotaxis, fitness

## Abstract

Microbial processes operate at the microscale, which is not resolved by existing ecosystem models. Here, we present a novel model that simulates a 1 mL three-dimensional cube using a hybrid Lagrangian–Eulerian approach, at ecologically relevant timescales. The model simulates individual microbes, including three phytoplankton size classes with healthy, senescent, and dead lifecycle stages; copiotrophic and oligotrophic heterotrophic bacteria; and dissolved organic matter at 50 µm resolution. Diffusion, shear, sedimentation, chemotaxis, and attachment processes are explicitly resolved. The emerging quantitative representation of the ecosystem shows that (1) copiotrophs grow mostly attached to eukaryotic phytoplankters and get almost all of their carbon from them vs. oligotrophs that grow on exudates and lysates of cyanobacteria; (2) contrasting diel patterns in substrate appearance in the phycosphere vs. ambient water and growth of particle-associated copiotrophs vs. free-living oligotrophs; (3) attached bacteria reduce carbon flux from the phycosphere, lowering chemotactic efficiency toward eukaryotes below that toward cyanobacteria; (4) shear reduces chemotactic efficiency and fitness of the copiotroph; and (5) the main benefit of chemotaxis is to locate attachment partners. These patterns are consistent with available observations. Our study provides insights into the microscale ecology of marine bacteria, and the open-source code is a tool for further research in this area.

**IMPORTANCE:**

A large amount of global CO_2_ fixation is performed by marine phytoplankton, and a substantial fraction of that is released as dissolved organic carbon and further processed by heterotrophic bacteria. The interaction between phytoplankton and bacteria, i.e., the carbon flux between them, is therefore an important process in the global carbon and climate system. Some bacteria have developed specialized behavioral traits, like swimming and attachment, to increase their carbon acquisition. These interactions occur at the micrometer scale, for example, the immediate vicinity of phytoplankters (the phycosphere), but existing biogeochemical models typically only simulate down to the 1 meter vertical or ~100 kilometer horizontal scale. We present a new microscale model and use it to predict fluxes and other features in the surface ocean. The model makes important predictions about the fluxes between various types of phytoplankton and bacteria and the role of behavioral traits, and it provides a basis and tool for further research in this area.

## INTRODUCTION

The surface ocean is a globally important microbial ecosystem playing a key role in climate, i.e., fixation of carbon and export to depth where it may be sequestered. The mechanistic, quantitative understanding of this complex system remains a grand challenge. Concentrations of the various components (phytoplankton, nutrients, etc.) can be readily observed (1–4). However, equally important are interactions and their quantitative description, e.g., resource flux, and the ecological role of various traits, e.g., the effect of chemotaxis on growth rate. These parameters are not easily observed in the field, and we are reliant upon models to resolve and quantify them (5–7).

Existing ecosystem models generally have relatively coarse spatial resolutions, e.g., 1 m vertical and ~100 km horizontal (5, 8). There are a number of reasons for that. First, computational costs of numerical models increase non-linearly with spatial resolution, i.e., the size and consequently number of computational segments. For example, reducing the layer depth twofold (e.g., from 1 to 0.5 m) while keeping the model extent constant translates into eightfold longer runtimes (9). Second, mechanistic understanding of processes operating at the microscale is still limited. Data used to calibrate and parameterize models come from measurements and laboratory experiments that traditionally use experimental/incubation volumes on the scale of liters [e.g., reference (10)]. Third, interest is often focused on bulk model output parameters that describe ecosystem services, like carbon export or primary productivity, which does not provide any motivation for increasing the resolution.

Contrary to the assumption of microscale homogeneity inherent in coarse-scale models, pelagic ecosystems are very heterogeneous at the microscale (11, 12). Individual phytoplankters release dissolved organic matter (DOM) via active and passive exudation and constitute point sources with high concentrations in their immediate vicinity, i.e., the phycosphere. Cellular lysis and grazing of phytoplankters result in near-instantaneous release of substrates at discrete locations, i.e., patches. The resulting concentration profile is spatially and temporally heterogeneous. Heterotrophic bacteria (hereafter simply referred to as bacteria) consume the DOM and further modify the concentration pattern.

Here, we are specifically concerned with the flux of resources, i.e., DOM, from phytoplankters to bacteria and the ecological role of various traits, e.g., chemotaxis, in this process. We consider two types of bacteria: copiotrophs and oligotrophs (13, 14). Copiotrophs, including e.g., *Roseobacter*, are larger, have more functions (including chemotaxis, attachment, and detachment and regulation), and grow fast in the presence of high substrate concentrations. Oligotrophs, including e.g., SAR11, are smaller, have fewer streamlined metabolic functions, and grow slow on low substrate concentrations. We ask: How much DOM flows between phytoplankters of various sizes (small, medium, and large) and lifecycle stages (healthy, senescent, and dead) to the copiotrophs and oligotrophs? What is the role of environmental factors (fluid shear) and transport traits (motility, chemotaxis, chemokinesis, and attachment) on the fitness of the bacteria?

Ecosystem models with explicit representation of one or more bacteria types have been developed, but those have been at a coarse spatial resolution (6, 7). Although copiotrophs and oligotrophs live in the same macroscale environment, the difference in transport traits (i.e., chemotaxis, attachment) means that they experience a much different microscale environment (15). Answering these questions therefore requires a microscale ecosystem model.

A number of microscale models have been developed previously (several are summarized in Table S1), which provided important insights into the microscale ecology of pelagic ecosystems. Most models were designed to explore interaction between chemotactic bacteria and substrate hotspots, and they generally have limited temporal extent (i.e., less than microbial generation time) and do not include many ecological mechanisms, like cell division or death. The model of Blackburn et al. (16) is an exception and includes many ecological features and longer temporal extent. However, that model also does not include several features deemed important here, like DOM exudation by phytoplankters and attachment by copiotrophs.

We developed a microscale model taking advantage of novel numerical integration methods and parallelized computing architecture. The model includes ecological mechanisms (e.g., cell division) and can be run at ecologically relevant timescales. We parameterize the model for the Bermuda Atlantic Time-series Study (BATS) site and quantify organic carbon flux from various phytoplankter classes to oligotroph and copiotroph bacteria and the ecological role of various traits in this process.

## MATERIALS AND METHODS

### Model overview

A detailed description of the model, including equations and parameters, is presented in the supplemental material, and a brief overview is presented here. The purpose of the model is to explore the ecology of bacteria growing on DOM produced by phytoplankton. It includes individual phytoplankters of three size classes, each with healthy, senescent, and dead (i.e., carcass, detritus) lifecycle stages, DOM, and individual copiotroph and oligotroph bacteria. The environment consists of a cube with periodic boundaries, e.g., microbes and chemical mass leaving at the right enter at the left. Individual microbes are simulated using an agent-based modeling approach, and DOM concentration is simulated using an Eulerian approach (17, 18). The model is numerical and divides space into discrete grid boxes (computational segments, cells). To present the bacteria with a continuous DOM concentration profile, we linearly interpolate the values for the bacteria. A simplified light-dark regime is applied.

The model includes diffusion of chemical and microbes and a velocity field to simulate fluid shear, i.e., dissipation of wind energy. The latter component is implemented in a simplified manner, as was done in other previous models (19, 20), which is dictated by the relatively small volume (computational constraints), and there is the need to avoid boundary effects and balance mass over long, ecologically relevant timescales, which is done using the periodic boundary conditions. Previous model analyses suggest that the shape of the velocity field (e.g., disk vs. tube) does not affect the bacterial distribution (21). Phytoplankters settle with a velocity depending on size and whether they are alive or dead (22). Copiotrophs perform run-reverse-flick chemotaxis and chemokinesis (i.e., higher velocity at higher substrate concentrations) based on previous models (23, 24) and attach and detach to and from phytoplankters (25, 26).

The phytoplankton population size is controlled by a simple carrying capacity approach (27), where a diel growth rate is specified and the death rate increases as the population size approaches the carrying capacity. The cell size increases with growth, and they divide into two daughter cells, using a slightly randomized biomass split fraction, once a threshold size is reached (17, 18). Healthy cells can become senescent at a specified rate. Death and senescence is implemented as a stochastic process (17). Phytoplankton cell death is assumed to be by zooplankton grazing, which is the dominant loss process for phytoplankton (28). Any bacteria attached to the grazed phytoplankter are killed, and most of the dead biomass is exported out of the model to deeper layers. DOM is produced by photosynthesis-proportional and basal exudation and phytoplankton death (lysis) events.

Bacteria grow on DOM using a simple Monod-type formulation that includes respiration and regulation. Substrate uptake is regulated, assuming transcriptional regulation for copiotrophs (longer activation time, stronger response) and post-translational regulation for oligotrophs (shorter activation time, weaker response), based on experimental evidence (15). Note that this model also predicts that transcriptional regulation is beneficial for chemotactic copiotrophs and not oligotrophs, consistent with our previous simpler model (15) (see Section S5.5 in the supplemental material). As for phytoplankton, bacteria divide when a threshold size is reached. Free-living cells die using a constant first-order lysis rate, whereas particle-associated bacteria are co-grazed with phytoplankters.

### Model parameterization

The model was parameterized to be representative of the mixed layer of stratified low-latitude open ocean surface waters, using observations from the BATS site (details in supplemental material). BATS metadata used in this model are deposited in the Biological and Chemical Oceanography Data Management Office (BCO-DMO) at http://lod.bco-dmo.org/id/dataset/861266, http://lod.bco-dmo.org/id/dataset/3782, and BATS website http://bats.bios.edu/bats-data/. The environment is a 1 mL cube with side dimensions of 1 cm. While computationally manageable (see below), the size of the model environment is sufficiently large to include about ~100 large phytoplankters (see below). The spatial resolution is 50 µm, which is of similar magnitude as the Batchelor scale [~10 µm, the smallest scale for nutrient gradients (29), note that concentration profiles are interpolated for bacteria; see above].

Three phytoplankton species representing different size classes, based on BATS core flow cytometry data (2), include small (SPHY, cyanobacteria, i.e., *Prochlorococcus + Synechococcus*, average diameter (*d*) = 0.73 µm, target cell concentration (*X*) = 4.1 x 10^4^ cells/mL), medium (MPHY, pico-eukaryotic phytoplankton, average *d* = 2.7 µm, *X* = 7.1 x 10^2^ cells/mL), and large (LPHY, nano-eukaryotic phytoplankton, average *d* = 4.7 µm, *X* = 1.0 x 10^2^ cells/mL). Phytoplanktons are parameterized to have a growth rate of ~0.5/d (30). The carrying capacity (see above) was set to the target concentration, and the base death rate (i.e., the death rate when the cell concentration is at the carrying capacity) was calibrated so that the cell concentration matches the carrying capacity. The fraction of biomass lost due to grazing was adjusted to produce enough substrate to support the target bacteria biomass (next paragraph).

Two bacteria “species” are included representing copiotrophs (COPIO, average *d* = 0.89 µm, *X* = 4.8 x 10^4^ cells/mL) and oligotrophs (OLIGO, average *d* = 0.52 µm, *X* = 4.3 x 10^5^ cells/mL). The classifications of copiotroph and oligotroph are pre-defined based on previous studies (13, 31, 32). The (target) cell concentrations are based on calculations of multiplying total bacterial abundance by specific amplicon sequence variants (ASVs) relative abundance (details in supplemental material). The oligotroph concentration is expectedly higher than observations for SAR11 [2.0 x 10^5^ cells/mL; Morris et al. (33)], because it includes other oligotrophs such as SAR86 and OM1 clade. The oligotrophic cell size is consistent with observations for SAR11 (34, 35), and a copiotroph/oligotroph cell biomass ratio of 5 was assumed. The corresponding total (target) bacteria biomass concentration is 400 μmolC/m^3^, consistent with that of Steinberg et al. (3). Oligotrophs have a lower maximum growth rate and half-saturation constant, consistent with growth in the free-living phase. Copiotrophs have higher values for those parameters and up-regulate and grow mostly in the attached phase. The total bacteria concentration is a function of the substrate source (see previous paragraph), and lysis and respiration parameters were adjusted to obtain relative copiotroph and oligotroph concentrations.

The model was calibrated to obtain phytoplankton and bacteria cell concentrations consistent with observations, as discussed above. Those model outputs are therefore “imposed” and not predictions *per se* (36). The new information obtained from the model, or “emergent properties,” includes the C fluxes, diel patterns, chemotactic efficiency, and fitness costs and benefits of various features, as presented in the Results section below. Those can be compared to independent estimates, for example, the C fluxes in Moran et al. (37).

The computation is challenging due to the large number of microbes and grid boxes and the small size of the latter, which requires a small timestep. We developed a computational approach suitable for parallelization, which divides the model volume into smaller sub-volumes and integrates them separately for a number of time steps (see supplemental material). The basecase simulation takes about 1.7 h wall time to compute 1 d model time on an HPC node with 192 logical processors.

### Quantifying benefit or cost of an environmental feature or bacterial trait

We use the model to quantify the benefit or cost to the bacteria of different environmental features and bacterial traits. Here, environmental features refer to mechanisms that affect all bacteria, like shear and phytoplankton sedimentation, and bacterial traits only directly affect specific bacteria, like chemotaxis, attachment and detachment, and regulation. The benefit or cost is quantified as a fitness coefficient (*f*), which is the normalized difference of net growth rates with and without the environmental feature or trait (*µ^+^*, *µ^−^*, 1/d), divided by the death rate (*k_L_*, 1/d) (*f* = [*µ^+^ – µ^−^*]/*k_L_*). Normalization to the death rate facilitates comparison of copiotroph and oligotroph cells that have different growth rates. When there is no difference in net growth rates, then *f* = 0, and when the difference is equal to the death rate, then *f* = 1.

For bacterial traits, we compare growth rates from strains with and without the trait in the same simulation. For environmental features, different simulations have to be used. That means, we compare growth rates from the basecase to a simulation where the feature is turned on or off during the simulation. This is subject to more stochastic differences (noise) between the simulations (e.g., one simulation may have several large phytoplankters die early releasing a burst of DOM), and therefore, less accurate and longer simulations are needed to average out the noise.

## RESULTS AND DISCUSSION

### Model testing and general features of results

We briefly point to some of the model testing and more general features of our results, which are discussed in more detail in the supplemental material. The model was tested against observations and other models for simplified scenarios, including 12 cases in total (see Section S4 in the supplemental material). This includes, for example, diffusion of DOM and microbes from an instantaneous point source and comparison to the analytical solution, chemotaxis of microbes toward a continuous point source (i.e., phytoplankter) and comparison to the model of Jackson (23), and chemotaxis of bacteria along a linear substrate gradient and comparison to the observations of Son et al. (24). The model was also used to simulate 30 laboratory mono- and co-culture experiments from five papers (32, 38–41), which shows that the model can reproduce the observed growth of bacteria on phytoplankton exudates. However, observations of antagonism, phytoplankton on bacteria and *vice versa*, were not reproduced by the model, as no such mechanism is included. Those patterns may also not be relevant under field conditions. For example, the phytoplankton *Emiliania huxleyi* is killed by the bacteria *Phaeobacter inhibens* in co-culture but not when other bacteria of the community are present (42).

In the full basecase simulation, the two bacteria types, copiotrophs and oligotrophs, stably co-exist in this spatially heterogeneous environment, which is consistent with previous experimental and theoretical research (see Section S5.1 in the supplemental material). The agent-based model simulates individual microbes, and the emergent distributions of experienced substrate concentration and growth rates and the emergent population size structure are heterogeneous (see Sections S5.2 and S5.3 in the supplemental material).

### A smaller illustrative simulation

**Fig 1:**
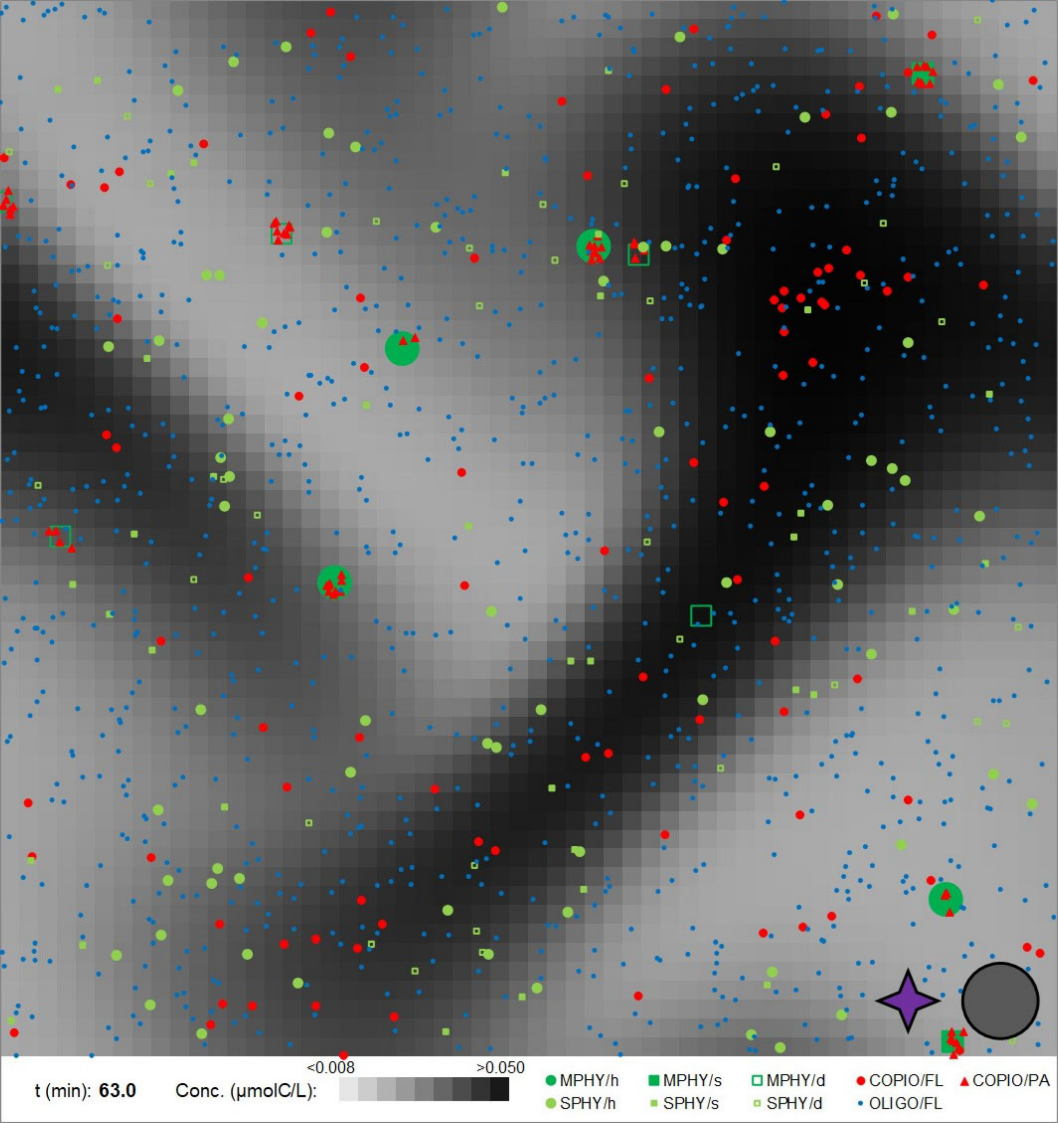
Model illustration. Plot dimensions are *x*, *y*, *z* = 1.4 mm and grid box = 25 µm. Microbes size shown exaggerated ×16. MPHY = medium phytoplankton, SPHY = small phytoplankton, h = healthy, s = senescent, d = dead, COPIO = copiotrophs, OLIGO = oligotrophs, FL = free-living, and PA = attached. Purple star symbol indicates shear rate. Gray circle indicates light. See Movie S1.

To illustrate some of the features of the model, we present results from a smaller simulation. For this small volume (1.4 mm cube), there is only an ~30% chance for a large phytoplankter to occur; thus, this simulation does not include one. We present a map (Fig. 1) along with long and short two-dimensional movies (Movie S1, 80 min, ×33 speed; Movie S2, ~1 min., real-time speed). Note that, although the three-dimensional simulations include realistic concentrations of microbes, the two-dimensional view also integrates the same distance “into the paper”; thus, microbes generally appear closer to each other than they actually are. We also included long and short three-dimensional movies (Movies S3 and S4) that look more realistic but do not clearly show all features (see below).

The map (Fig. 1) shows attachment of copiotrophs to healthy, senescent, and dead medium-sized phytoplankters. A (relatively) large DOM patch, originating from grazing (sloppy feeding) of a healthy medium phytoplankter a couple of minutes before the time of the figure, is occupied by copiotrophs. The patch is modified by diffusion and shear, and it also illustrates the periodic boundary condition.

The long movie (Movie S1) illustrates several ecologically relevant features:

- Shear (throughout movie). The magnitude of shear is indicated by the purple symbol in the lower right-hand corner of the video. Note that velocity also varies with distance “into the screen”; thus, while cells may appear to be in the same location that they are actually advected in different directions.
- Sedimentation of medium phytoplankters (throughout movie). Other microbes do not gravitationally settle at appreciable velocities. Carcasses are shown as open symbols and they sediment at faster rates.
- DOM diffuses out from small phytoplankters and attracts copiotrophs (mostly from carcasses, most visible early in the simulation when the background concentration is low).
- Death of phytoplankters produces new DOM patches, and copiotrophs readily move to and occupy them before they are dispersed (throughout movie). Note that the time step of the long movie is too coarse to resolve the run-reverse-flick chemotaxis (copiotrophs appear to “jump”); however, this trait is illustrated in the shorter movie.
- Senescence of a medium phytoplankter and “hitchhiking” of attached copiotrophs (20 min)
- Division of a medium phytoplankter and “vertical transmission” of attached copiotrophs (28 + min)
- Death of a medium phytoplankter results in the production of a DOM patch and attraction of copiotrophs (at simulation time 61 min; see also Fig. 1).

The short movie includes death of a small phytoplankter that results in the production of a DOM patch and chemotaxis of a copiotroph toward that patch (Movie S2). Corresponding traces of two representative cells illustrate advection superimposed by a random walk of the oligotroph and run-reverse-flick chemotaxis of the copiotroph (Fig. S6).

### Carbon flows from eukaryotes to copiotrophs and cyanobacteria to oligotrophs

**Fig 2:**
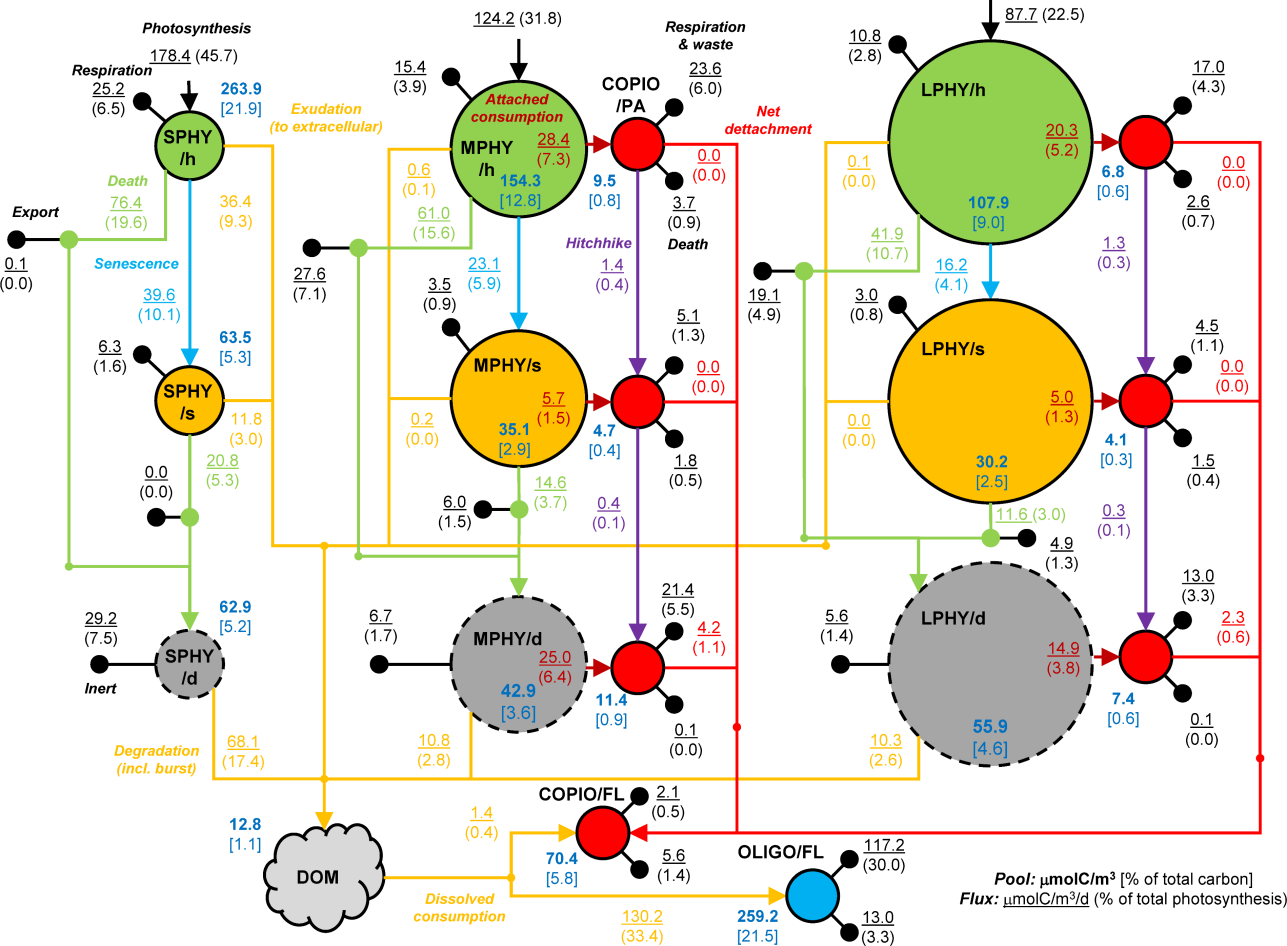
Carbon fluxes through and within the ecosystem. SPHY = small phytoplankton, MPHY = medium phytoplankton, LPHY = large phytoplankton, h = healthy, s = senescent, d = dead, COPIO = copiotrophs, OLIGO = oligotrophs, FL = free-living, PA = attached, and DOM = dissolved organic matter. Numbers: pool size in μmol C/m^3^/d and percentage of total in square brackets (blue text); fluxes in μmol C/m^3^/d and percentage of total photosynthesis in parentheses (various colors). Sedimentation loss is usually a minor flux (e.g., 0.9 µmol C/m^3^/d for LPHY/d) and included in death or inert for clarity. Small imbalances in fluxes result from stochastic population fluctuations.

Stores and fluxes of carbon are summarized in Fig. 2. To illustrate, we describe the stores and fluxes for healthy medium phytoplankters in detail. Their concentration is 154.3 µmol C/m^3^ and that of their attached copiotrophs is 9.5 µmol C/m^3^. Photosynthesis produces 124.2 µmol C/m^3^/d of which 15.4 µmol C/m^3^/d is respired, 28.4 µmol C/m^3^/d is consumed by attached bacteria, 0.6 µmol C/m^3^/d is released to the extracellular environment, 61.0 µmol C/m^3^/d is lost to grazing (27.6 µmol C/m^3^/d of that is exported, the remainder is converted to carcasses and released as DOM, i.e., sloppy feeding, included in carcass “burst” in figure), and 23.1 µmol C/m^3^/d is lost as cells transition from healthy to senescent phase of growth. The model is at an approximate steady state, so there is no net flux going into the biomass, i.e., growth. Of the 28.4 µmol C/m^3^/d consumed by the bacteria, 23.6 µmol C/m^3^/d is respired and wasted, 1.4 µmol C/m^3^/d is transferred (i.e., hitchhike) as their healthy host becomes senescent, 3.7 µmol C/m^3^/d is grazed, and net attachment adds 0.038 µmol C/m^3^/d (to re-colonize bacteria-free phytoplankter offspring).

**Fig 3:**
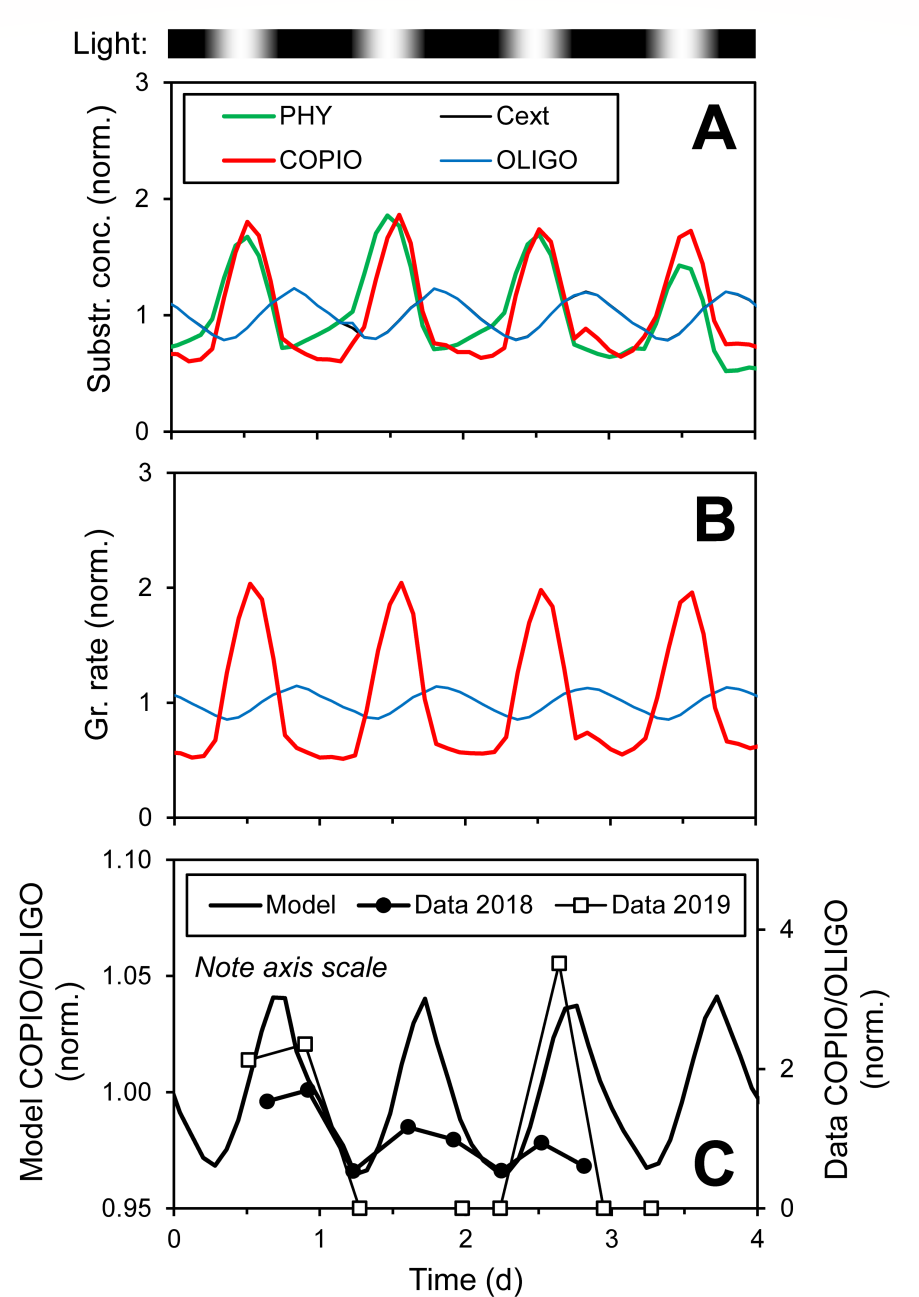
Diel pattern. (**A**) Substrate concentration at phytoplankton surface, i.e. phycosphere (PHY, average of all size and life stages), ambient medium (Cext), and experienced by copiotroph (COPIO) and oligotroph (OLIGO) cells. Note blue and black lines are very close and barely distinguishable. (**B**) Growth rate of bacteria. (**C**) Ratio of COPIO to OLIGO cell concentrations. All values normalized to mean.

Several of the modeled C fluxes can be compared to a recent estimate (37, see Table 1 therein) (Fig. 3). Of the net primary production (photosynthesis – respiration), 26% is released as DOM (from healthy cells, exudation, attached consumption), which is consistent with the estimate of 25% (dissolved primary production). Release from senescent cells (exudation, attached consumption) is 7%, which is also consistent with the estimate of 6% (lysis, senescence). Grazing (burst) releases 7%, which is lower than the estimate of 18% (sloppy feeding, egestion). This is expected since much of the egestion of diel-migrating zooplankton occurs at depth, and in our model of the surface ocean, this is considered a sink, i.e., vertical export. Bacterial production (equal to death at steady state) is 9% of net primary production, consistent with the estimate of 12% (bacterial production).

The copiotrophs get almost all (99%) of their substrate while attached to medium and large (eukaryotic) phytoplankters. Of the DOM produced by those phytoplankters, most (91%) is consumed by attached copiotroph cells and the rest (9%) “escapes” to the extracellular DOM pool, where it constitutes a small fraction (8%) of the C input. The fraction of the extracellular DOM pool “standing stock” coming from medium and large phytoplankters is slightly larger (10%), presumably because the larger patches are not consumed as efficiently. The oligotrophs consume exclusively from the extracellular DOM pool; thus, 90% of the DOM source is from small (cyanobacteria) phytoplankters. We were unable to find empirical observations that speak toward this conclusion. However, stable isotope tracing or NanoSIMS experiments may be able to provide further evidence for these fluxes (43, 44).

Free-living copiotroph cells are mostly down-regulated (see Materials and Methods and Section S5.2 in the supplemental material), have a small DOM consumption and a negative net growth rate. The free-living copiotroph population size is maintained by net detachment from dead phytoplankton cells.

### Copiotrophs and oligotrophs show different diel patterns

Photosynthesis and exudation follow a diel pattern, which is reflected in the substrate concentration on the phytoplankter surface (i.e., phycosphere) and the concentration experienced by copiotroph cells (Fig. 3). The ambient medium concentration, i.e., the concentration experienced by the oligotroph, is less variable and expectedly lags the phytoplankter surface concentration (note blue and black lines are very close and barely distinguishable in figure). This lag and difference in amplitude is also reflected in the growth rates. Consequently, the relative cell concentrations of copiotrophs and oligotrophs also show a diel pattern.

Observations from two diel surveys at BATS are consistent with these model results (Fig. 3C). Here, ASVs classified as extreme oligotrophs and strong and extreme copiotrophs by reference (13) are used (see Section S3.2. in the supplemental material for details). Those observations show a much stronger amplitude than the model, which may be due to the selection of strong and extreme copiotrophs, for which a larger fraction may be particle associated. These model results are also consistent with *in situ* transcriptomic observations from other sites, which showed that, in general, metabolic genes for SAR11 (free-living) peaked later in the light period than *Roseobacter* (phytoplankton-associated) (45–47).

### Chemotactic efficiency toward cyanobacteria but not eukaryotes

**Fig 4:**
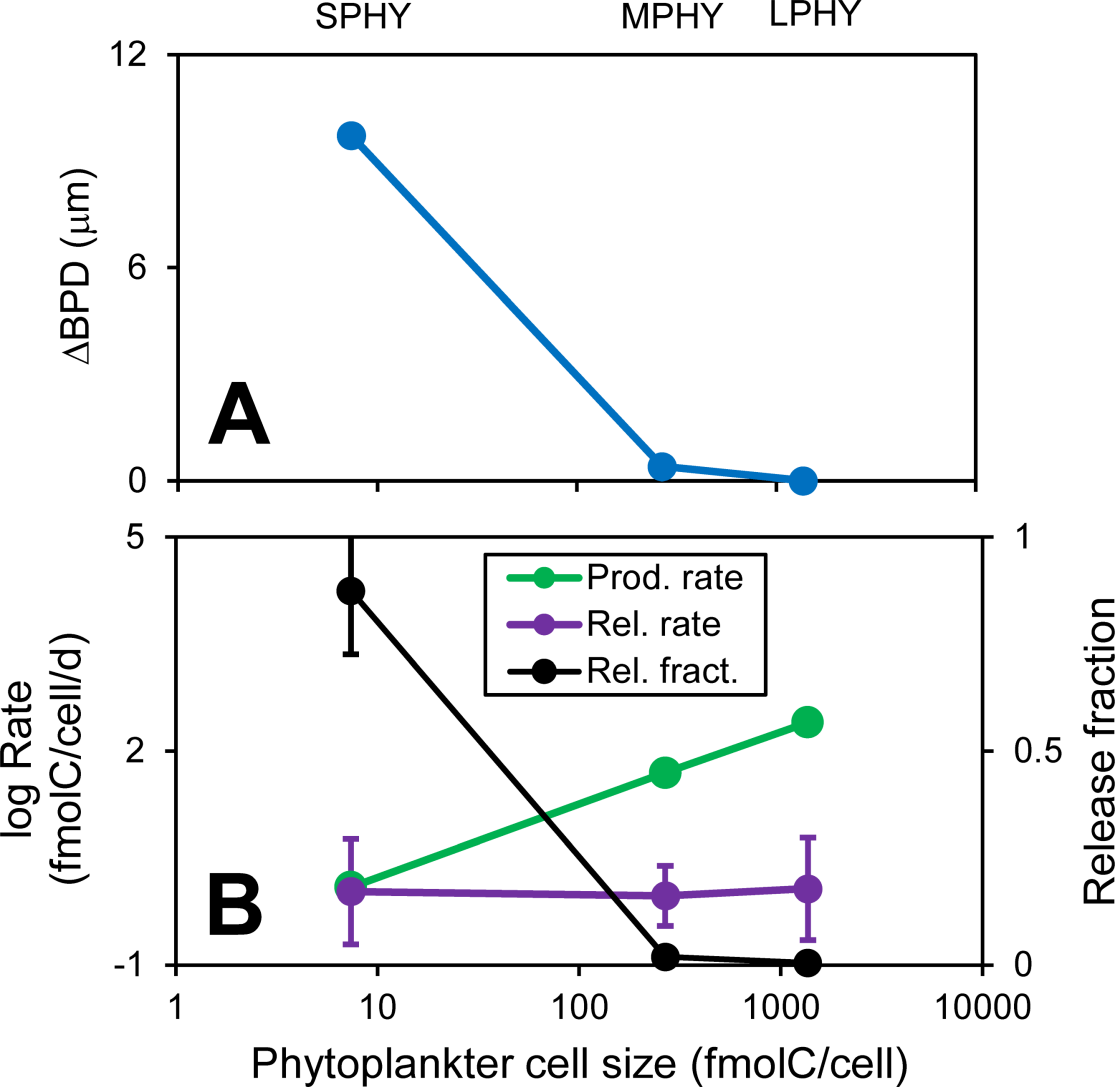
Effects of phytoplankter cell size on chemotactic efficiency and phycosphere organic carbon release. (**A**) Relative bacteria-phytoplankter distance (BPD; ΔBPD = oligotroph BPD – copiotroph BPD). (**B**) Organic matter production and release rates for phytoplankters and corresponding release fraction. Only healthy phytoplankters are included. Error bars are ±1 SD.

To quantify the chemotactic efficiency, we first calculate the average distance from (free-living) bacteria to the nearest phytoplankter (bacteria-phytoplankter distance, BPD). Then, to remove the effect of reproductive clustering [reference (19); see Section S5.4 in the supplemental material], we calculate the difference between the BPD of chemotactic copiotrophs and non-chemotactic oligotrophs, expressed as ΔBPD. This value quantifies how much closer copiotrophs are to phytoplankters compared to oligotrophs. The model predicts chemotaxis toward small phytoplankters (ΔBPD = 10 µm), but not toward medium and large phytoplankters (Fig. 4A). Chemotaxis toward cyanobacteria (small phytoplankton in our model) has been observed (48). The lack of chemotaxis toward the medium and large phytoplankters is counter to the expectation that larger sources make for more attractive chemotaxis targets. However, those cells are further apart and the local chemical structure, which drives chemotaxis, is driven by the small phytoplankters (including senescent and dead). Further, consumption by attached bacteria reduces the organic carbon flux out of the phycosphere for the larger phytoplankters (Fig. 4B; see also mass balance above). Note that this does not apply to axenic phytoplankters (can occur following division of medium phytoplankters), which are colonized rapidly by bacteria in the model, where chemotaxis is essential (see below). We are not aware of any observational evidence to support or refute this model prediction and further experiments might provide evidence to confirm this result. Blocking substrate flux out of phycospheres may also have ecological implications, e.g., related to microbiome assembly, priority effects, and turnover.

### Shear and sedimentation lower chemotactic efficiency and costs copiotrophs

**Fig 5:**
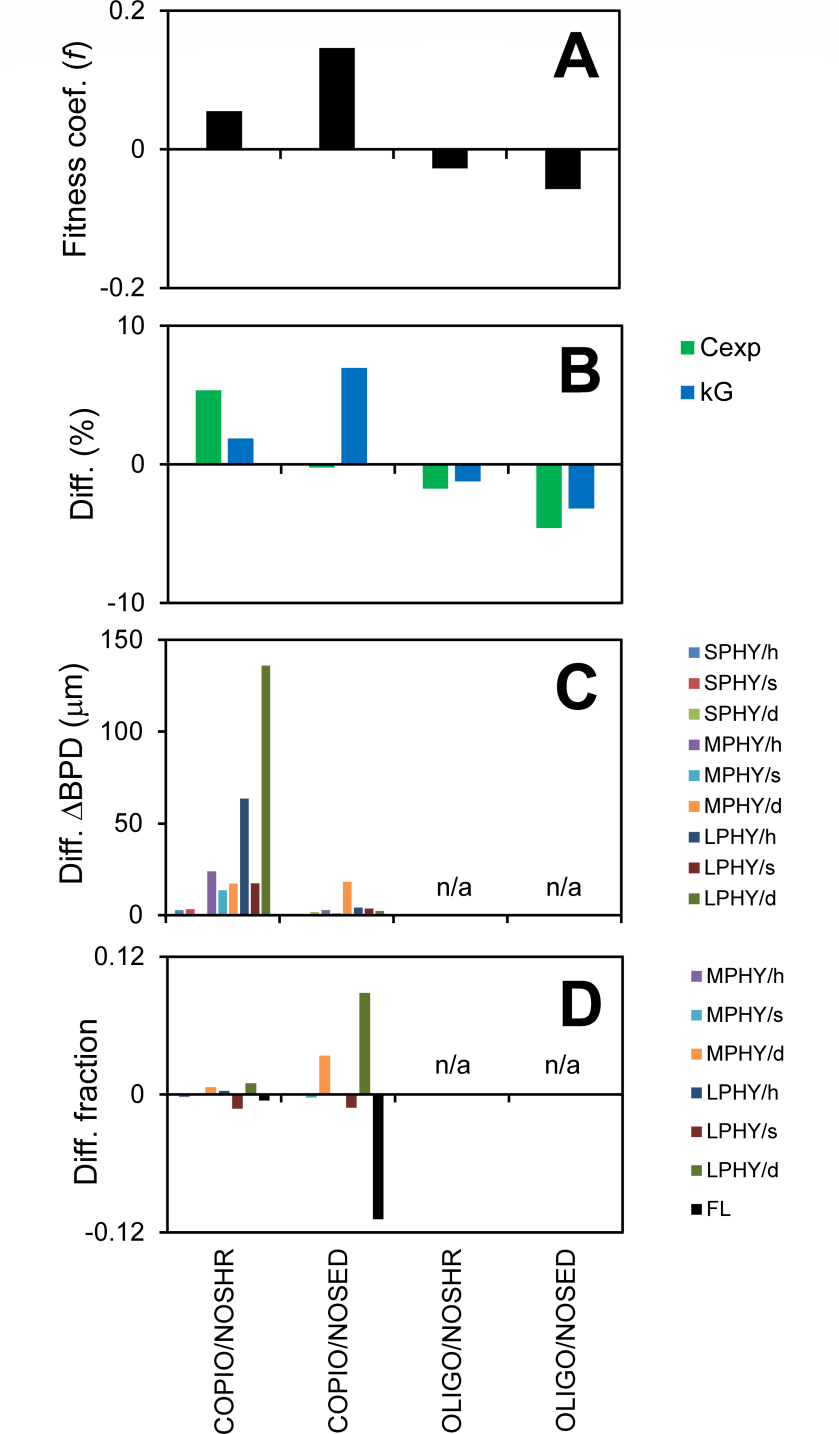
Effect of shear and phytoplankton sedimentation on bacteria fitness. (**A**) Normalized growth rate difference (fitness coef., *f*). (**B**) Relative change in substrate concentration experienced (Cexp) and growth rate (kG). (**C**) Change in chemotactic efficiency (ΔBPD). (**D**) Change in particle-associated and free-living fractions (e.g., MPHY/d is difference in fraction attached to medium, dead phytoplankters). SPHY = small phytoplankton, MPHY = medium phytoplankton, LPHY = large phytoplankton, h = healthy, s = senescent, d = dead, COPIO = copiotrophs, OLIGO = oligotrophs, NOSHR = simulation without shear, and NOSED = simulation without phytoplankton sedimentation.

To investigate the cost or benefit of shear and phytoplankter sedimentation to bacteria, we take the basecase (which includes those processes), turn them off or increase them during the simulation and compare growth rates. The results show that shear and sedimentation cost the copiotroph and benefit the oligotroph. Results for removing these processes are presented here (Fig. 5A) and those for increasing them, which show consistent results, are presented in the SI (Fig. S33).

Shear may affect bacteria fitness via a number of mechanisms. First, shear can reduce the chemotactic efficiency by “sweeping bacteria away from phytoplankters” [(Bowen et al. (21); experiments also reproduced with the present model, see Section S4.8 in the supplemental material], which is supported by several published predictions and observations (49). Second, shear may increase nutrient flux to cells by compressing the diffusion boundary layer, although theoretical analysis suggests this process is not significant for small cells (50). Note that our model has a 50 µm resolution so it does not resolve this scale around the ~1 µm bacteria. Third, shear can “stir nutrient patches into networks of filaments that motile bacteria can readily exploit” (29), in essence increasing the surface area of the patch. Our model with the simplified shear velocity field also generates nutrient filaments that are localized by and feed copiotrophs (see Fig. 1; Movie S1). Fourth, shear reduces reproductive clustering of phytoplankters (see Table S22) and the bacteria-phytoplankter distance (BPD) even without chemotaxis.

In the model, when shear is removed, the chemotactic efficiency of the copiotroph increases, especially for dead large phytoplankters (Fig. 5C). The net effect is an increase in concentration experienced, growth rate, and fitness. Removing shear may also reduce DOM availability (see previous paragraph), but since the concentration experienced goes up, the increased chemotactic efficiency outweighs that effect. The more efficient consumption of the copiotroph reduces the overall substrate concentration in the ambient phase (−1.6%), which is reflected in the reduced concentration experienced by the oligotroph. Thus, without shear, the oligotroph experienced reduced growth rate and fitness.

Removing sedimentation also benefits the copiotroph, albeit by a different mechanism. Without sedimentation, attachment to dead large (and medium) phytoplankters increases (Fig. 5D). Note that the sedimentation velocity of dead large phytoplankters is about 4.9 µm/s, which is of comparable magnitude as the chemotactic velocity (the net upgradient velocity) of 14 µm/s. More bacteria in this “active” up-regulated phase increases the population growth rate. More attached cells reduce the phycosphere substrate concentration of dead large phytoplankters (−48%), which also slightly reduces the average concentration experienced by the population. By attaching a larger fraction of their population, the copiotrophs make more efficient use of the available substrate, which lowers the substrate concentration. Note that reducing sedimentation loss also increases the concentration of the faster-settling large dead phytoplankters, but that effect is small (+3.7%).

### Main benefit of chemotaxis is to find attachment partner

We use the model to quantify the benefit of motility (m), chemotaxis (c), chemokinesis (k), and attachment (a) for the copiotrophs by competing strains with and without these features and computing a fitness coefficient (*f*; see Methods). In the basecase simulation, the copiotroph generally down-regulates its metabolism, and does not grow in the free-living phase (see Section S5.2 in the supplemental material), which would not be a meaningful strategy for a non-attachment lifestyle. We therefore turn off regulation for these simulations. Costs for motility (propulsion), chemotaxis and chemokinesis (sensing), and attachment (production of extracellular polymeric substances) are reflected in respiration rate and yield parameters (details in supplemental material).

**Fig 6:**
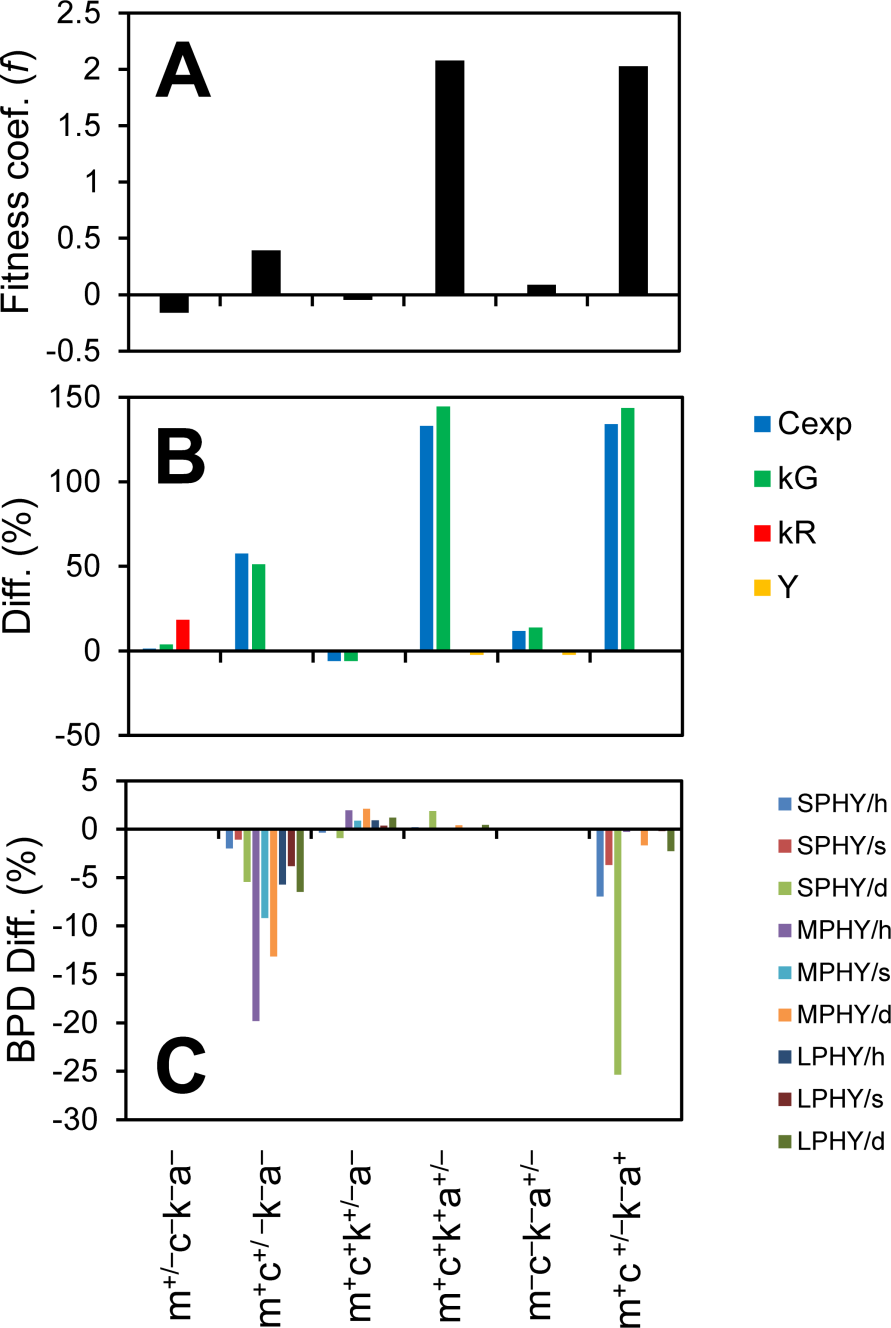
Effect of motility (m), chemotaxis (c), chemokinesis (k), and attachment (a) on copiotroph fitness. (**A**) Normalized growth rate difference (fitness coef., *f*). (**B**) Change in substrate concentration experienced (Cexp), growth and respiration rates (kG, kR), and yield (Y). (**C**) Change in bacteria-phytoplankter distance (BPD). Values shown are for the strain with the trait compared to the one without the trait, e.g., the strain with chemotaxis has lower BPD values. SPHY = small hytoplankton, MPHY = medium phytoplankton, LPHY = large phytoplankton, h = healthy, s = senescent, and d = dead.

A competition between copiotrophic strains with and without motility (m^+^c^–^k^–^a^–^ vs. m^–^c^–^k^–^a^–^) shows that motility is not beneficial, mostly due to the higher respiration rate (Fig. 6). However, the motile strain does experience a slightly higher substrate concentration and growth rate because it continually moves toward “new pastures.” Swimming has been suggested as a mechanism to overcome diffusion limitation (51), and some marine bacteria have motility but no chemotaxis genes (52, 53). Also, recent experiments showed a slightly higher (not statistically significant) substrate uptake for motile vs. non-motile (both non-chemotactic) cells (48) (see Fig. 2B of reference; lower cyanobacteria concentrations, corresponding to stronger diffusion limitation). Note that the model does not resolve the sub-grid scale drawdown of substrate, meaning it likely underestimates the benefit of motility.

Competing strains with and without chemotaxis (m^+^c^+^k^–^a^–^ vs. m^+^c^–^k^–^a^–^) shows a benefit of this trait. Chemotaxis brings bacteria closer to phytoplankters, which increases the experienced substrate concentration and growth rate. A competition between strains with and without chemokinesis (m^+^c^+^k^+^a^–^ vs. m^+^c^+^k^–^a^–^) shows that chemokinesis reduces the chemotactic efficiency, although the effect is small and does not change fitness. Competing strains with and without attachment (m^+^c^+^k^+^a^+^ vs. m^+^c^+^k^+^a^–^) show that attachment provides a large benefit, due to higher experienced substrate concentration and growth rate (Fig. 6).

The analysis above considers the benefit of individual traits, but there is also interdependence among the traits. Specifically, attachment is not very beneficial without motility, as shown by a competition between non-motile strains with and without attachment (m^–^c^–^k^–^a^+^ vs. m^–^c^–^k^–^a^–^; Fig. 6). Also, a competition between chemotactic and non-chemotactic strains of copiotrophs, both with attachment (m^+^c^+^k^–^a^+^ vs. m^+^c^–^k^–^a^+^), shows a much larger benefit to the chemotactic strain than the corresponding competition without attachment. These results suggest that the main benefit of chemotaxis is not to increase the substrate concentration experienced in the free-living phase but to locate attachment partners, i.e., chemotaxis enables attachment, a result consistent with experimental observations (26, 54, 55).

It is well known that ecological traits are not independent, but that there exist correlations, connections, and tradeoffs (56). A trait tradeoff generally means that as one trait becomes more beneficial another trait becoming less beneficial. For example, nutrient affinity is sacrificed as maximum uptake velocity increases (57). Another type of dependence, which we will call “trait prerequisite,” is where one trait becomes beneficial only if another trait is present. For example, previous work suggests that transcriptional regulation is only beneficial for motile cells because they stay in patches long enough to up-regulate uptake via this mechanism (15). In fact, there are several traits clustering with the copiotrophic and oligotrophic lifestyles (13). Here, there are obvious prerequisites, like chemotaxis and chemokinesis requiring motility, but also less obvious, like chemotaxis aiding attachment.

### Summary and outlook

Here, we present a novel microscale ecology model that simulates the substrate interaction of bacterial copiotrophs and oligotrophs with phytoplankton in the surface ocean ecosystem. The simulations show that (1) copiotrophic bacteria predominantly grow on and obtain their substrate from eukaryotes (medium and large phytoplankters) and oligotrophs obtain substrate from exudates and lysates of cyanobacteria (small phytoplankters), (2) substantial diel patterns with a time-lagged appearance of substrate in the ambient environment (vs. phycosphere) and corresponding lag in growth of oligotrophs (vs. copiotrophs), (3) lower chemotactic efficiency to larger phytoplankters because attached cells reduce the substrate flux out of the phycosphere, (4) shear and phytoplankter settling reduce chemotactic efficiency and growth of the copiotroph, and (5) the main benefit of chemotaxis is to aid attachment (vs. the higher concentrations in the vicinity of phytoplankters).

The model is a first step toward mechanistic modeling of microscale interactions between phytoplankton and bacteria in the surface ocean. The open-source code can be used as is to explore the ecology of other ecosystems like coastal or inland pelagic surface waters. Note that organic carbon input in the model is from local primary production only; thus, the model in its current configuration is suitable for exploring questions in the surface mixed layer only.

There are many possible extensions to the model. The number of microbial species could be increased and viruses or membrane vesicles (58–60) could be added. However, for species with lower cell concentrations a larger environment would need to be simulated to include (i.e., sample) sufficient agents. The number of interacting chemical compounds could be readily increased. For example, DOM substrates could be separated into high and low molecular weight sizes with varying diffusivity, i.e., larger molecules with lower diffusion as substrates for copiotrophs and *vice versa*, production and function of cell-free and -bound exoenzymes (61, 62), bacteria-produced substrates, allelopathic chemicals, and other extracellular chemicals (e.g., oxygen, reactive oxygen species) (63–66). This would require more complex representation of intracellular metabolism (67).

Previous work and the results of this study suggest that microscale processes are important drivers of microbial ecology. Given that microbes play an important part in the biogeochemical processes, it is critical to incorporate these microscale processes into our large, ecosystem-scale models (7). At this scale, it is not possible to explicitly resolve individual microbes or the chemical field at the microscale, and simplified computational approaches will need to be developed.

## SUPPLEMENTAL MATERIAL

Supplemental Information mbio.02372-24-s0001.docx Supplemental text, tables, and figures.

Movie Legends mbio.02372-24-s0002.docx Legends for supplemental movies.

Movie S1 mbio.02372-24-s0003.mp4 Long two-dimensional movie, model illustration.

Movie S2 mbio.02372-24-s0004.mp4 Short two-dimensional movie, model illustration.

Movie S3 mbio.02372-24-s0005.mp4 Long three-dimensional movie, model illustration.

Movie S4 mbio.02372-24-s0006.mp4 Short three-dimensional movie, model illustration.

ASM does not own the copyrights to Supplemental Material that may be linked to, or accessed through, an article. The authors have granted ASM a non-exclusive, world-wide license to publish the Supplemental Material files. Please contact the corresponding author directly for reuse.
