# Supplementary material for: Emergent ecology in a microscale model of the surface ocean
Source: mBio. 2024 Oct 9;15(11):e02372-24. doi: 10.1128/mbio.02372-24 (PMC11559031; doi:10.1128/mbio.02372-24)
Supplement: Supplemental Information — Supplemental text, tables, and figures. [file mbio.02372-24-s0001.docx]

**Supplementary Materials for**

Emergent ecology in a microscale model of the surface ocean

Eigemann, et al.

correspondence to: [ferdi.hellweger@tu-berlin.de](mailto:ferdi.hellweger@tu-berlin.de)

**Contents:**

S1. Summary of pelagic microscale microbial models

S2. Model details

S3. Model parameterization

S4. Model testing

S5. Additional model results

S6. References

# Summary of pelagic microscale microbial models

1. Summary of pelagic microscale microbial models

| **Model**  **Reference** | **Microbes** | | | **Che.**  **Typ. (a4)** | **Tran.**  **(a5)** | **Space** | | | **Time**  **(d)** |
| --- | --- | --- | --- | --- | --- | --- | --- | --- | --- |
|  | **Types**  **(a1)** | **N**  **(a2)** | **Proc.**  **(a3)** |  |  | **Dim.**  **(a6)** | **Res.**  **(μm) (a7)** | **Ext.**  **N/V(mL)**  **(a8)** |  |
| [Jackson (1)](#_ENREF_1)  (c1) | B(1) | 3.2e3 | c(rt) | D(1) | D | 3D | inf. | -/4.2e-6 | 1.2e-3 |
| [Bowen et al. (2)](#_ENREF_2)  (c1) | B(1) | 1.0e3 | c(rt) | D(1) | D, T | 3D | inf. | -/1.0e-3 | 2.8e-2 |
| [Blackburn et al. (3)](#_ENREF_3) | P(1),  B(1),  Z(1) | 1.0e3 | p, h(m), z, c(bd), d | D(1), N(1) | D | 2D | 130 | 4.9e3/9.8e-3 | 2.1e1 |
| [Taylor and Stocker (4)](#_ENREF_4) | B(2) | C | h(d), c(v) | D(1) | D, T | 3D | 110 | 1.3e8/1.8e2 | 1.4e-3 |
| [Son et al. (5)](#_ENREF_5)  (c1) | B(1) | 3.0e3 | c(rrf), k | D(1) | D | 2D | inf. | -/9.0e-4 | 8.1e-2 |
| [Brumley et al. (6)](#_ENREF_6) | B(1) | 2.0e3 | c(rrf) | D(1) | D | 3D | inf. | -/1.8e-3 | 3.5e-3 |
| [Christensen et al. (7)](#_ENREF_7) | M(2) | 1.0e5 | c(gt) | - | T | 3D | 830 | 1.9e9/1.1e5 | 6.9e-4 |
| [Noell et al. (8)](#_ENREF_8) | B(2) | 1.0e5 | c(rrf) | D(1) | D | 3D | inf. | -/8.0e0 | 1.4e-2 |
| [Raina et al. (9)](#_ENREF_9) | B(3) | 5.0e2 | c(rrf) | D(1) | D | 3D | inf. | -/2.5e-1-2.5e-3 | ? |
| This model  (b1) | P(h,s,d)(1),  B(2) | 6.0e5 | p, h(r), g, m, c(rrf), k, d, e, a, s | D(1), N(1) | D, S | 3D | 50 | 8.0e6/1.0 | ~1.0e2 |

(a1) P = phytoplankton (h = healthy, s = senescent, d = dead), B = bacteria, Z = grazer, M = microbe (generic)

(a2) Number included in model. C = concentration

(a3) Proc. = Process; p = photosynthesis, h = heterotrophy (m = Monod, r = regulation, s = storage, d = diffusion-limited), g = growth & division, m = motility, c = chemotaxis (bd = biased diffusion, rt = run & tumble, rrf = run-reverse-flick, gt = gyrotaxis, v = chemotactic velocity), k = chemokinesis, d = death, e = exudation, a = attachment, s = sedimentation, z = grazing

(a4) Che. Typ. = Chemical Types; D = DOM, N = inorganic nutrient

(a5) Trans. = Transport; D = diffusion, S = shear, T = turbulence

(a6) Dim. = Dimension.

(a7) Res. = Resolution; inf. = infinitesimal, i.e. analytical solution.

(a8) Ext. = Extent. N = number of grid boxes (“-“ indicates no spatial discretization, i.e. analytical solution), V = total volume.

(b1) Corresponds to basecase simulation; experiments and test cases have additional features (e.g. competition between two types of copiotrophs); inorganic nutrients are implicitly considered using carrying capacity approach.

(c1) Direct comparison to our model presented in the SI.

# Model details

## Overview

This section presents the details of the model. A standard protocol for describing individual-based and agent-based models, the ODD (Overview, Design concepts and Details) protocol, is used to facilitate understanding and duplication of the model ([10](#_ENREF_10)). The ODD protocol consists of seven elements, grouped into overview (purpose; entities, state variables, and scales; process overview and scheduling), design concepts and details (initialization; input data; submodels).

## Purpose

The purpose of the model is to explore the spatial ecology of copiotrophs and oligotrophs at the microscale. Specifically, we aim to quantify the benefit of traits like chemotaxis, attachment to phytoplankters, regulation, etc.

## Entities, state variables, and scales

The low-level entities in the model include grid boxes with chemical mass and individual microbes. Microbe state variables are listed in Table S2. The model environment is a cube divided into grid boxes. The time step is limited by numerical stability. The temporal extent varies among the experiments and ranges from one minute to 100 days.

1. Grid box and microbe state variables

| **Name** | **Units** | **Description** |
| --- | --- | --- |
| Grid box |  |  |
| *C* | µmol/L | Chemical concentration |
| Microbe |  |  |
| *ia* | - | Index |
| *im* | - | Species ID (e.g. SPHY, OLIGO, …) |
| *sn* | - | Serial number |
| *on* | - | Lifecycle ID (e.g. healthy, senescent, …) |
| *x, y, z* | m | Location |
| *θ*, *ϕ* | rad. | Orientation |
| *m* | molC/cell | Size |
| *CIP* | molC/m^3^ |  |
| *C* | µmolC/L | Chemical concentration |
| *Cprev* | µmolC/L | Chemical concentration at previous timestep |
| *vmoti* | µm/s | Base speed |
| *dv* | - | Direction (forward or backward) |
| *adPbdt* | 1/d | (a) |
| *iaP* | - | Index of phytoplankter attached to |
| *Q* | molC/molC | Internal stored substrate |
| *t_R_* | min. | Regulation time |
| *f_A_* | - | Regulation activation factor |
|  |  |  |

(a) Running, exponentially decaying average of the rate of change of the fraction of bound surface receptors. See Eq. (17).

1. Phytoplankton lifecycle stages (A) and biomass (B).

## Process overview and scheduling

The model proceeds in discrete time steps. After initial set up (e.g. create initial microbe population), at each time step, the following steps are performed:

1. Write output
2. Update shear velocity field
3. Calculate chemical advection
4. Calculate chemical diffusion and update concentration
5. Update microbe carrying capacity
6. Calculate microbe kinetics: Shear(a), diffusion and sedimentation, chemotaxis, boundary, heterotrophy, photosynthesis, exudation, growth, lysis, carrying capacity
7. Update microbe array (e.g. remove dead agents, create new agents)
8. Advance time

(a) To avoid spatial disconnect between microbes and chemical field, microbe shear needs to be done with same velocity field as preceding chemical shear.

Modified for parallelization and batch stepping, see S2.9.b.

## Design Concepts

### Basic principles

The model simulates extracellular substrate as a chemical concentration and individual microbial cells as agents.

### Emergence

The dynamic population size and behavior, including growth rate, emerge from the cumulative action and interaction of the agents. For example, the average distance for COPIO to MPHY is (BPD, Table 1 in the main paper) is an emergent property.

### Adaptation

Individual cells respond extracellular substrate concentration by changing growth (incl. regulation) and chemotaxis. Bacteria also attach to phytoplankter cells when they touch and the phytoplankter surface concentration is high enough.

### Objectives

The adaptive behavior of the cells is not based on the explicit optimization of some objective. Rather, the behavior of the cells is designed to reproduce observed behavior from laboratory experiments, as already incorporate in existing and established sub-models (e.g. Monod growth, chemotaxis). These are optimized by evolution to maximize the fitness of the species.

### Learning

The model does not include learning.

### Prediction

The model does not include prediction.

### Sensing

Cells sense the concentration of extracellular substrate at their location, light level and their own states. Spatial gradients in chemical concentration are sensed as temporal gradients by the cells, which is used for chemotaxis. Bacteria sense phytoplankters within attachment threshold distance.

### Interaction

Cells interact with each other indirectly via extracellular substrate. Bacteria attach to phytoplankter cells and consume organic carbon directly from their phycospheres.

### Stochasticity

Stochasticity is included in number of processes, including diffusion, cell death, lysis and division (at division biomass of daughter cells is varied from the ideal 50/50 split).

### Collectives

The model does not consider collectives (i.e. the formation of colonies). However, a number of bacteria may attach to a phytoplankter and are transported with the phytoplankter.

### Observation

The model saves the statistics of the population (e.g. average cell size) and the properties of an individual cell at specified time intervals. It also saves the properties of the entire population at specified times.

## Initialization

The model can be initialized with chemical and microbial population created from average properties specified in the input, or it can use the values from a previous simulation as a starting point (i.e. hot start, checkpointing).

## Input data

The model uses parameter values as input data (listed in the corresponding sections below), and otherwise does not utilize any input.

## Submodels

Here, each process is described in detail. Model parameters are defined along with the equations for each process below. Literature values for parameters (where available) are listed in the corresponding tables.

### Initial conditions

For chemicals, the initial concentration is specified. The value can be varied within a range to add some initial heterogeneity. For microbes, the initial concentration is specified. The microbes are generally placed randomly throughout the model domain (except for in some test cases, see Section S4) with random orientation, size and other states.

### Boundary conditions

The model generally uses a periodic/wrapping boundary condition. For model testing, the code also supports radiating and assigned/fixed boundary conditions. For the assigned/fixed boundary condition, microbes are lost when they reach the boundary.

### Photosynthesis, respiration, exudation and senescence

*Photosynthesis and respiration*

Photosynthesis is simulated as a first-order process with a fixed assigned rate (*k_P,MAX_*, 1/d). That is, the phytoplankton concentration is controlled using a carrying capacity approach, as described in Section S2.8.g. Photosynthesis is distributed across the diel cycle, using zero at nighttime (before 6:00 and after 18:00) and a half sine curve at daytime. A constant respiration rate is used (*k_R_*, 1/d).

*Exudation*

Exudation occurs at a constant exudation fraction of photosynthesis (*f_E_*) plus a basal exudation rate (*k_E,b_*, 1/d). A constant fraction of the exudate is considered bioavailable (*f_a_*). See Fig. S2 for an illustration.

The model tracks an intracellular exudate concentration (*C_I,P_*, mol/L). This concentration changes due to production of exudate, membrane diffusion and/or active exudation (out only, added to the extracellular substrate pool) and growth dilution, as well as consumption by attached bacteria (discussed in Section S2.8.l). The mass balance is:

$$V_{cell}\frac{dC_{I,P}}{dt}=\left( k_{P} f_{E}+k_{E,b} \right) f_{a} m-k_{m}A_{cell} C_{I,P}-k_{G}C_{I,P} V_{cell}$$

where *k_m_* (m/s) is the membrane permeability/transport coefficient. The surface area (*A_cell_*, m^2^) and volume (*V_cell_*, m^3^) are calculated from the cell biomass (*m*, molC/cell) assuming a spherical shape.

*Senescence and host killing*

Healthy cells may enter a senescent state, where they do not perform photosynthesis (see Fig. S1). Entry to this stage is stochastic based on a first-order rate (*k_sen_*, 1/d). Senescent cells do not synthesize new biomass and their size gradually decreases due to respiration and exudation. The cell dies and transitions to a carcass when the size drops below a threshold (*m_sen,X_*, molC/cell). In addition, extrinsic death (i.e. grazing) may also kill senescent cells (see Section S2.8.g). The exudation rate and membrane transport coefficient changes by a factor for senescent cells (*f_E,s_*, *f_km,s_*).

Attached bacteria may kill senescent cells by increasing the exudation rate (*f_E,s,b_*), which decreases the time it takes to reach the size threshold (*m_sen,X_*). This option is not used in the simulations presented in the paper.

1. Photosynthesis & exudation parameters

| **Name** | **Units** | **Value** | **Notes** |
| --- | --- | --- | --- |
| *k_P,MAX_* | 1/d | SPHY: 0.70  MPHY: 0.80  LPHY: 0.80 | (a)  0.69 – 3.6, μmax, various species, [Di Toro (11)](#_ENREF_11).  1.0 – 2.0, μmax, model range, [Schnoor (12)](#_ENREF_12).  0.75 – 1.0, μmax, various species, [Morel (13)](#_ENREF_13) (b).  0.2 – 2.0, range used in model, [Weitz et al. (14)](#_ENREF_14).  1.0 – 2.5, range summarized by [Hellweger and Lall (15)](#_ENREF_15). |
| *k_R_* | 1/d | 0.10 | 0.001 – 0.1, range used in model, [Weitz et al. (14)](#_ENREF_14).  0.01 – 0.25, range summarized by [Hellweger and Lall (15)](#_ENREF_15). |
| *k_sen_* | 1/d | 0.15 | (a)  0.03 – 0.31, range used in model, [Painting et al. (16)](#_ENREF_16). |
| *m_sen,X_* | molC/cell | = 0.5 × *m_0_* | - |
| *f_E_* | - | 0.20 | 0.13, literature summary, [Baines and Pace (17)](#_ENREF_17).  0.25, literature summary, [Moran et al. (18)](#_ENREF_18).  0 − 0.70, range of literature values, [Connolly et al. (19)](#_ENREF_19). |
| *k_E,b_* | 1/d | 0.033 | 0.005 – 0.1, range used in model, [Weitz et al. (14)](#_ENREF_14).  0.1-0.2, respiration and exudation, [Chapra (20)](#_ENREF_20). |
| *f_E,s_* | - | 5 (c) | - |
| *f_E,s,b_* | - | 1 | Not used. |
| *f_a_* | - | 1.0 | - |
| *k_m_* | m/s | 5.0e-12 | Set to get about 10% exudate mass fraction. |
| *f_km,s_* | - | = *f_E,s_* | - |
|  |  |  |  |

(a) See Section S3.4.

(b) Calculated using Eq. 4 in Table 1 of reference.

(c) Set to get factor ~1 senescent / healthy total exudation, see main text. Calculation based on same cell size, i.e. at enter senescence. Average exudation of senescent cells will be lower due to smaller size. *f_E,s_* × *k_E,b_* = *k_P,MAX_* × *f_E_* + *k_E,b_*.


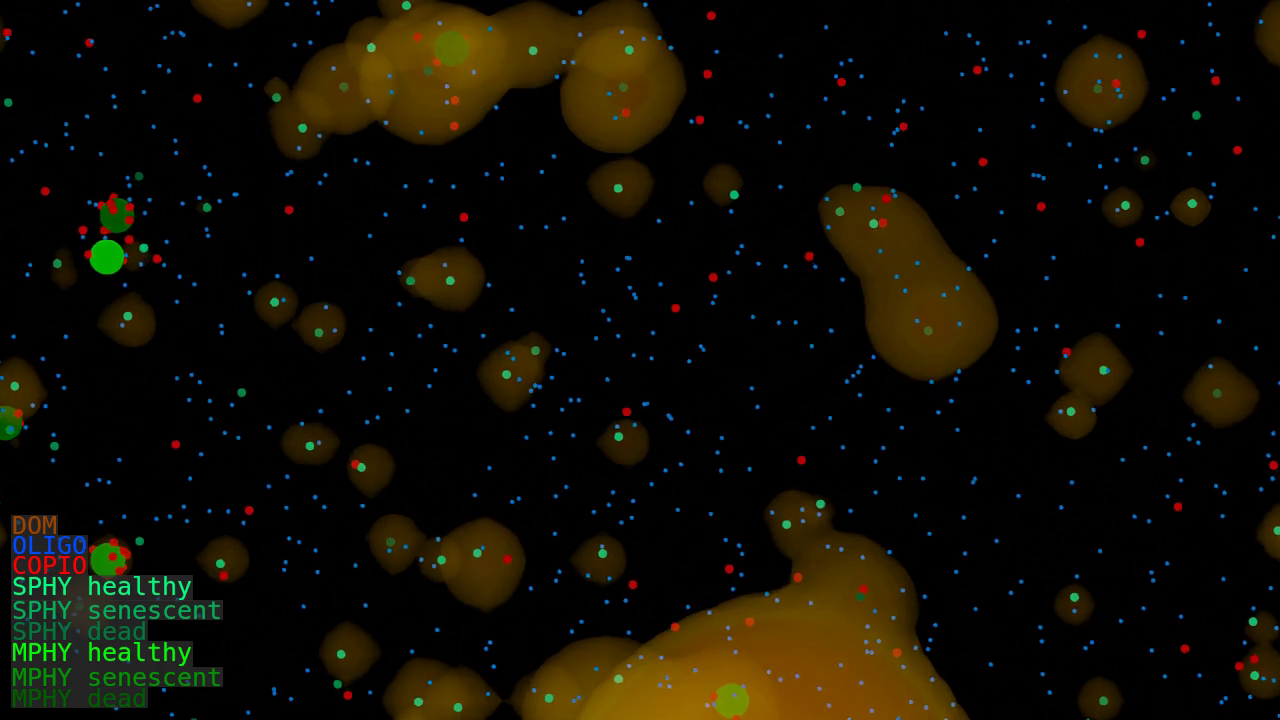


1. Illustration of exudation. Plot dimensions are x, y, z = 1.4 mm, 0.8 mm, 1.4 mm. Microbes size shown exaggerated x16. DOM = Dissolved organic matter (stronger color corresponds to higher concentrations), OLIGO = oligotrophs, COPIO = copiotrophs, SPHY = small phytoplankton, MPHY = medium phytoplankton. See Movie S4.

### Heterotrophy

Heterotrophic growth is simulated using a basic Monod equation, and modifications to include regulation, storage or both (depending on the case evaluated).

*Basic*

For basic heterotrophic growth, a Monod formulation with the max. growth rate proportional to the specific surface area (calculations based on a spherical cell shape) is used:

$$k_{G}=k_{G,MAX}\frac{C}{K_{S}+C}=k_{G,MAX,AVE}\left( \frac{m}{m_{AVE}} \right)^{-\frac{1}{3}}\frac{C}{K_{S}+C}$$

where *k_G,MAX_* (1/d) is the max. growth rate, *K_S_* (mol/m^3^) is the half-saturation constant and *C* (mol/m^3^) is the extracellular substrate concentration experienced by the cell. *m* (molC/cell) is the cell size, and *k_G,MAX,AVE_* (1/d) and *m_AVE_* (molC/cell) are the corresponding values for the average cell size. The substrate consumption rate is a function of the yield coefficient (*Y*) as described in Section S2.8.m.

Respiration is modeled as a first-order rate (*k_R_*, 1/d). To simulate dormancy and avoid mini-cells, respiration is turned off when the cell size is below a threshold (*m_D_*, see Table S7). However, in the simulations presented here this threshold is rarely reached. Net biomass growth is based on the growth minus respiration rates (see next section).

1. Heterotrophy/basic parameters

| **Name** | **Units** | **Value** | **Notes** |
| --- | --- | --- | --- |
| *k_G,MAX,AVE_* | 1/d | See Table S8 | 0.35 – 1.3, range summarized by [Mentges et al. (21)](#_ENREF_21) (a).  0.18 – 0.72, range used in model, [Weitz et al. (14)](#_ENREF_14) (a).  0.65 – 4.7, range used in model applications, [Connolly et al. (19)](#_ENREF_19).  0.65 – 1.4, range of literature values, [Bucci et al. (22)](#_ENREF_22) (a).  1.7, Delaware coastal waters, [Kirchman (23)](#_ENREF_23) (a).  0.40 – 17, predicted from codon usage bias, [Long et al. (24)](#_ENREF_24).  0.065 – 100, [Vieira-Silva and Rocha (25)](#_ENREF_25). |
| *K_S_* | μmolC/L | See Table S8 | 0.043 – 0.38, range used in model applications, [Bucci et al. (22)](#_ENREF_22).  1.7 – 6.7, range used in model, [Weitz et al. (14)](#_ENREF_14) (b).  ~6.0 *E. coli* ABC transporters (c).  4.4 – 17 (d).  6 – 450, range summarized by [Mentges et al. (21)](#_ENREF_21).  17 – 1,500, range used in model applications, [Connolly et al. (19)](#_ENREF_19). |
| *Y* | . | 0.20 | 0.05 – 0.2, range used in model, [Weitz et al. (14)](#_ENREF_14).  < 0.01 – 0.32, BGE for freshwater isolates, [Muscarella et al. (26)](#_ENREF_26).  < 0.05 – 0.6, BGE for natural planktonic bacteria, [Giorgio and Cole (27)](#_ENREF_27).  0.20 – 0.52, range used in model applications, [Connolly et al. (19)](#_ENREF_19).  0.09 – 0.33, range summarized by [Mentges et al. (21)](#_ENREF_21).  0.8 – 0.9, high quality substrates, [Connolly et al. (19)](#_ENREF_19).  See Section S3.6 for adjustments due to no motility etc. |
| *k_R_* | 1/d | See Table S8 | 0+ (e)  0.001 – 0.1, range used in model, [Weitz et al. (14)](#_ENREF_14).  0.07 – 0.15, range used in model applications, [Connolly et al. (19)](#_ENREF_19).  See Section S3.6 for adjustments due to no motility etc. |
|  |  |  |  |

(a) Converted using *Y* = 0.36.

(b) Converted using 0.15 molN/molC (Redfield).

(c) Based on “*K_m_* values of *E. coli* ABC transporters for sugars are in the range of 1 µM” [Ihssen and Egli (28)](#_ENREF_28), converted using 180 g/mol and 6 molC/mol.

(d) Based on “most high affinity uptake systems have *K_m_* values in the range of 100 – 500 µg L^-1^ for sugars” [Egli (29)](#_ENREF_29).

(e) Respiration constitutes consumption of cell biomass. This can be as low as zero considering basal metabolism and chemotaxis can be supported by rhodopsins ([30](#_ENREF_30), [31](#_ENREF_31)).

*Diffusion limitation*

In reality, substrate consumption by the cell leads to a draw-down of concentration in the vicinity of the cell, the bacteria sense the concentration at the cell surface (used for chemotaxis, regulation, growth, …), and the concentration gradient drives a diffusive flux towards the cell. This is a dynamic scenario with feedback (more growth > lower concentration > less growth > higher concentration > more growth > …). For the extreme case of zero concentration at the cell surface (a “perfect absorber”) and steady state, the flux can be calculated and sets an upper bound on the growth rate ([32](#_ENREF_32)), which is commonly referred to as “diffusion limitation”.

In the model, the growth rate is parameterized based on the grid box-average substrate concentration and the sub-grid scale drawdown around the cell is not explicitly considered. However, to account for diffusion limitation, which is expected to be important for bacteria under many conditions (i.e. low substrate concentrations), a max. uptake/growth rate is applied:

$$k_{G,DL}=\frac{Y}{m} \left( 4 \pi D_{C} r C \right)$$

where *Y* is the yield, *m* (molC/cell) is the cell size, *D_C_* (m^2^/d) is chemical diffusion coefficient, *r* (m) is the cell radius and *C* (molC/m^3^) is the grid box chemical concentration. The term in parentheses is Eqn. 2.21 in [Kiørboe (32)](#_ENREF_32).

The diffusion-limitation constraint does not apply to attached cells.

Note that depression of substrate concentration due to consumption also happens at the grid scale (see Fig. 6 and associated discussion, experiments with and without motility).

The figure below illustrates the diffusion-limitation constraint. For the copiotroph, the up-regulated growth rate is generally above the diffusion-limitation, whereas the down-regulated is below that. Since the copiotroph is generally down-regulated in the free-living phase, diffusion-limitation does not play a role there. It is up-regulated in the particle-attached phase, but there the diffusion-limitation constraint does not apply. For the oligotroph, the growth rate is close to diffusion-limitation at lower substrate concentrations, and below that at higher substrate concentrations.

1. Diffusion-limitation. Growth rates of copiotroph (up- and down-regulated) and oligotroph for various substrate concentrations.

*Regulation*

Regulation is modeled in a simplified binary manner by turning the growth (or uptake, if storage is used) rate down or up. We differentiate between “regulation” as the intent and “activation” as the success.

The system is generally upregulated when the concentration is above a threshold (*C_R_*). In addition, it is up-regulated when the cell is attached to a phytoplankter. This is done to avoid presumably unrealistic cycling when the surface concentration drops below the regulatory threshold, which happens when carcasses become very small. Specifically, the feedback loop *lower surface concentration > down-regulation > lower consumption > higher surface concentration > up-regulation > lower surface concentration > …* leads to repeated up and down-regulation.

When the system is upregulated, the regulation time counter (*t_R_*, min.) increases and once it reaches an activation time threshold (*T_A_*, min.) the system is on. When the concentration is below the threshold, the regulation time counter decreases and when it reaches zero the system is off. On/off switching is done by multiplying the max. growth (or uptake, if storage is used) rate (Eq. (2)) by a binary switch (*f_A_* = *f_A,on_* or *f_A,off_*).

To account for the different metabolic costs when the system is on and off, the respiration rate is modified. Specifically, the total respiration is split into a base component that is not affected by the regulatory status and a component that accounts for the metabolic cost when the system is on:

$$f_{R,R}=\left( 1-r_{A} \right)+r_{A} f_{A}$$

where *f_R,R_* is the time-variable respiration factor for regulation (applied to *k_R_* in Eq. (8)) and *r_A_* is the variable fraction of the respiration associated with the growth system. This is following the approach of [Hellweger (33)](#_ENREF_33), although there *r_A_* accounts for assimilation of one substrate up to a specific intracellular pool (*Q*), whereas here it accounts for the entire assimilation and growth system.

1. Heterotrophy/regulation parameters

| **Parameter** | **Units** | **Value** | | **Notes** |
| --- | --- | --- | --- | --- |
|  |  | Transcr.  (COPIO) | Post-transl.  (OLIGO) |  |
| *C_R_* | µmolC/L | 0.1 | 0.001  (always on) | See Section S3.5. |
| *T_A_* | min. | 5 | 0.5 | (c) |
| *f_A,off_* | - | 0.05 | 0.5 | (c) |
| *f_A,on_* | - | 1 | 1 | By definition. |
| *r_A_* | - | 0.9 | 0.1 | = 0.9 corresponds to 10% basal maintenance respiration.  = 1 corresponds to complete down-regulation. |

(c) Time and magnitude parameters based on [Noell et al. (8)](#_ENREF_8).

1. Regulation: Illustration of method. Time series of a copiotroph encountering attaching a MPHY cell. (A) DOM experienced and regulation threshold concentration (*C_R_*). (B) Regulation time counter (*t_R_*) and activation time threshold (*T_A_*). (C) Regulation on/off switch (*f_A_*). (D) Growth rate (*k_G_*).

*Storage*

Note that storage is included in the model, although it is not used in any of the simulations presented in the main paper.

Storage is modeled by including an intracellular stored substrate pool (*q*, molC/molC), and explicitly simulating uptake and growth ([33-35](#_ENREF_33)). Uptake is simulated using a Monod-type model with the max. rate proportional to the surface area (see above), and a limitation term based on *q*:

$$V=V_{MAX}\frac{C}{K_{M,C}+C} \frac{q_{MAX}-q}{q_{MAX}}=V_{MAX,AVE}\left( \frac{m}{m_{AVE}} \right)^{-\frac{1}{3}}\frac{C}{K_{M,C}+C} \frac{q_{MAX}-q}{q_{MAX}}$$

The corresponding consumption rate for extracellular substrate is thus *V* / *Y* *m* (molC/cell/d) (see Section S2.8.m). The conversion rate from intracellular stored substrate to biomass (i.e. the growth rate) is modeled via a Monod-type model:

$$k_{G}=k_{G,MAX,AVE}\frac{q}{K_{M,q}+q}$$

The mass balance for intracellular stored substrate is:

$$\frac{dq}{dt}=V-k_{G}-\left( k_{G}-k_{R} \right)q$$

The last term accounts for growth dilution.

1. Heterotrophy/storage parameters

| **Parameter** | **Units** | **Value** | **Notes** |
| --- | --- | --- | --- |
| *V_MAX,AVE_* | molC/molC/d | *=* *k_G,MAX,AVE_* × *K_M,C_* / *K_S_* | (a) |
| *K_M,C_* | µmolC/L | = *m_AVE_* × *f_bst_* × *f_a_* / *V_box_* for LPHY | (a) |
| *q_MAX_* | molC/molC | 100 | (a) |
| *k_G,MAX,AVE_* | molC/molC/d | - | See Table S4 |
| *K_M.q_* | molC/molC | 1.0 | (a) |

(a) Storage parameters were set based on the assumption that the uptake system does not saturate (*K_M,C_* = max. exp. conc.), and that at steady-state and at very low *C* and *C* = *K_S_*, the growth rate is the same as without storage (sets *V_MAX,AVE_* and *K_M,q_* based on Eqn. (5) - (7), with *C*, *K_S_* << *K_M,C_*, *q* << *q_MAX_*). The max. internal stored substrate level (*q_MAX_*) was set at a relatively high value to allow for substantial storage. See Section S4.11.

### Cell growth

The cell size (*m*, fmolC/cell) is quantified on a biomass C per cell basis, where biomass C includes structural biomass, and excludes intracellular exudate (phytoplankton, see Section S2.8.c) or stored substrate (bacteria, see Section S2.8.d). The cell size increases with growth:

$$\frac{dm}{dt}=\left( k_{P}-k_{R}-k_{P} f_{E}-k_{E,b} \right) m or \left( k_{G}-k_{R} \right) m$$

### Cell division

Cells divide when their size reaches a threshold (*m* ≥ 2 *m_0_*) ([36](#_ENREF_36)). For phytoplankton, a time-of-day division gate is implemented (*t_C,D,a_*, *t_C,D,b_*, d) ([37](#_ENREF_37), [38](#_ENREF_38)). To account for phenotypic variability, the size is varied from the ideal 50/50 split fraction by drawing from a truncated (to avoid unrealistic values, e.g. split fraction > 1) normal distribution ([39](#_ENREF_39), [40](#_ENREF_40)).

1. Cell division parameters

| **Name** | **Units** | **Value** | **Notes** |
| --- | --- | --- | --- |
| *m_AVE_* | molC/cell | See Table S8 | - |
| *m_0_* | molC/cell | = *m_AVE_* / 2 / ln(2) | ([36](#_ENREF_36)) (a) |
| *R_CV_* | - | 0.1 | Typically ~0.1 ([41](#_ENREF_41)) |
| *m_D_* | molC/cell | = 0.5 × *m_0_* | Dormancy cell size, see Section S2.8.d. |
| *t_C,D,a_, t_C,D,b_* | h | 6, 18 | End, start of division gate  Division at night, *Prochlorococcus* ([38](#_ENREF_38), [42](#_ENREF_42)) |
|  |  |  |  |

(a) Theoretical relationship between *m_AVE_* and *m_0_* in continuous culture, *m_AVE_* will be higher when division gate is used, ~×1.2, based on model results.

1. Strain-specific parameters

| **Strain** | ***m_AVE_***  **(molC/cell)** | ***r_AVE_***  **(μm)** | ***D_M,AVE_***  **(m^2^/s)** | ***ρ_C_***  **(fgC**  **/μm^3^)** | ***k_G,MAX,AVE_***  **(1/d)** | ***K_S_***  **(µmolC**  **/L)** | ***k_R_***  **(1/d)** | ***k_L_***  **(1/d)** | ***K***  **(μmolC**  **/L)** |
| --- | --- | --- | --- | --- | --- | --- | --- | --- | --- |
| COPIO | 3.0e-15 (a1) | 0.45 (e1) | 5.5e-13 (c1) | 97 (d1) | 50 (d) (d5) | 10 (d) (d4) | 0.1 (c) | 0.08 (c) | - |
| OLIGO | 6.0e-16 (a1) | 0.26 (e1) | 9.4e-13 (c1) | 97 (d1) | 0.30 (d) | 0.025 (d) | 0.05 (c) | 0.05 (c) | - |
| SPHY | 5.6e-15 (a3) | 0.37 (e1) | 6.7e-13 (c1) | 325 (e2) | - | - | - | - | 0.23 (a3) |
| MPHY | 1.9e13 (a3) | 1.3 (e1) | 1.8e-13 (c1) | 229 (e3) | - | - | - | - | 0.14 (a3) |
| LPHY | 9.9e-13 (a3) | 2.4 (e1) | 1.0e-13 (c1) | 214 (e3) | - | - | - | - | 0.10 (a3) |
|  |  |  |  |  |  |  |  |  |  |

(a1) See Section S3.2

(a3) Based on cell counts (cells/ml) and/or C concentration (mgC/m^3^), BATS Core Data, MLD.

(e1) Calculated from *m_AVE_* assuming spherical shape and *ρ_C_*.

(e2) From [DuRand et al. (43)](#_ENREF_43).

(e3) Using log *m_AVE_* (pgC/cell) = 0.96 log *V_AVE_* (μm^3^) – 0.6 as in [DuRand et al. (43)](#_ENREF_43).

(c1) Based on *r_AVE_*. See Section S2.8.h.

(d1) Based on 1.1 gWW/mL and 0.22 gDW/gWW ([44](#_ENREF_44)), and 0.4 gC/gDW.

(d2) *m_AVE_* / *V_AVE_* (from *r_AVE_* assuming sphere).

(c) For OLIGO: Regulation not applicable (system is always on), *k_G_* = *k_L_* + *k_R_* at steady-state. For COPIO: regulation reduces *k_R_* in FL phase, *k_L_* = 0 and grazing loss in PA phase.

(d4) Set high, above max. phytoplankter surface concentration, i.e. assume no saturation.

(d5) Set low enough to avoid reducing healthy phytoplankter surface concentration below detachment concentration.

(d) See main text and Section S3.4.

### Extrinsic death

The model includes an extrinsic death process that accounts for external factors, like zooplankton grazing and viral lysis. Sedimentation loss is described in Section S2.8.j. Senescence of phytoplankton is an intrinsic death process and is described in Section S2.8.c. Extrinsic death is evaluated stochastically ([45](#_ENREF_45)).

*Bacteria*

For bacteria, a constant first-order death rate (*k_L_*, 1/d) is specified, which is applied to free-living cells. Extrinsic death (i.e. grazing) of phytoplankters is also a loss of the attached bacteria, i.e. they are removed from the model.

*Phytoplankton*

For phytoplankton, a carrying capacity approach is used ([46-48](#_ENREF_46)). Here, the death rate (*k_K_*, 1/d) is:

$$k_{K}=k_{K,b} \left( \frac{MC}{K} \right)^{n_{K}}$$

where *k_K,b_* (1/d) is the base death rate (corresponding to *MC* = *K*), *K* (μmolC/L) is the carrying capacity, *MC* (μmolC/L) is the biomass concentration of healthy (i.e. resource-consuming) cells and *n_K_* is an exponent. For small phytoplankton, a constant rate ([42](#_ENREF_42)) and no export is used, whereas for medium and large phytoplankton the rate is applied only at nighttime consistent with diel zooplankton migration/grazing, and a fraction (*f_exp_*) is exported.

Upon death, the phytoplankton agent is marked as a carcass/detritus and continues to be tracked by the model. After a fraction of the mass is exported, the remainder consists of three pools (see Fig. S1B), including fixed unavailable mass (*m_car,X_*, molC/cell), variable unavailable mass ((*m* – *m_car,X_*) × (1 – *f_a_*), molC/cell), and variable available mass ((*m* – *m_car,X_*) × *f_a_*, molC/cell). The unavailable mass is assumed to be refractory at the timescale of the simulation. Initially, a fraction of the variable mass is released (“burst fraction”, *f_bst_*). Then, the remaining variable mass is “leaked” from the carcass. The process is assumed to be mediated by enzymes at the carcass surface and an area-based rate is used (*k_car_*, molC/m^2^/d).

1. Death parameters

| **Name** | **Units** | **Value** | **Notes** |
| --- | --- | --- | --- |
| *k_L_* | 1/d | See Table S8 | - |
| *k_K,b_* | 1/d | SPHY: 0.24  MPHY: 0.30  LPHY: 0.30 | (a1) |
| *K* | μmolC/L | See Table S8 | Target concentration, corresponds to *X* (cells/ml) in main paper. |
| *n_K_* | - | 2 | - |
| *f_bst_* | - | 0.10 | (b)  = 0.025-0.40, based on [Mine et al. (49)](#_ENREF_49) |
| *f_exp_* | - | 0.50 | (a2) |
| *k_car_* | molC/m^2^/d | 0.0043 | = 0.0043, corresponds to *k_LPOM_* = 0.5/d for MPHY at *m_AVE_*. (b1)  *k_LPOM_* (1/d):  = 0.033-0.15, based on [Mine et al. (49)](#_ENREF_49)  = 0.07-0.1, labile POM hydrolysis rate (d)  = 0.5, POC hydrolysis rate (e) |
| *m_car,X_* | molC/cell | 0.1 × *m_0_* | (b) |
|  |  |  |  |

(a1) Calibrated to get phytoplankton *MC* = *K*.

(a2) Calibrated to get bacteria *MC* = target concentration, see Section S3.4.

(b1) *k_LPOM_* = *k_car_* × *A_cell_* / *m_AVE_.*

(b) The carcass will exist for a time ≈ ln( *m_car,X_* / [ *m_AVE_* × ( 1 – *f_bst_* ) ] ) / *k_LPOM_*.

(d) Literature range and value used in model by [Hellweger and Lall (15)](#_ENREF_15).

(e) [Grossart and Simon (50)](#_ENREF_50), as cited by [Takeshi and Norio (51)](#_ENREF_51).

### Diffusion

For diffusion of extracellular substrate, see Section S2.8.m. Microbes diffuse using a stochastic process ([45](#_ENREF_45)). For example, the change in *x* (m) position in a time step (*Δt*, d) is:

$$\Delta x=ran \sqrt{2 D_{M} \Delta t}$$

where *ran* is a random number drawn from a standard normal distribution and *D_M_* (m^2^/s) is the microbe diffusion coefficient. The diffusion coefficient is a function of the individual’s cell size (*r*, radius), calculated as in [Lambert et al. (52)](#_ENREF_52) (*D_M_* = *k* *T* / ( 6 π *η* *r* ), *k* = 1.38e-23 m^2^ kg / s^2^ / °K, *T* = 25°C, *η* = 0.89 mPa s).

1. Diffusion parameters

| **Parameter** | **Units** | **Value** | **Notes** |
| --- | --- | --- | --- |
| *D_C_* | m^2^/s | 1.0e-10 (p)  *ave. D_C,n_* = 1.9e-11 (n) | = 1e-12 – 1e-9 (a)  = 3.2e-12 – 3.2e-9 (b)  = 1e-10 (c)  = 1e-9 (d)  = 1e-9 (e)  = 1e-10 (f) |
| *D_M,AVE_* | m^2^/s | See Table S8 | - |

(a) Solutes consumed by marine bacteria, [Taylor and Stocker (4)](#_ENREF_4).

(b) Molecules with intermediate diffusivities, [Smriga et al. (53)](#_ENREF_53).

(c) [Blackburn et al. (3)](#_ENREF_3).

(d) [Jackson (1)](#_ENREF_1), Fig. 3 and Table 1.

(e) [Bowen et al. (2)](#_ENREF_2).

(f) Corresponds to diffusion limited growth of OLIGO with *k_G_* = 0.1 1/d at *C* = 0.01 μmolC/L, see Eqn. (3), Parameters in Table S4, Table S8.

(p) See Section 0.

(n) Numerical dispersion, see Section S2.8.i.

### Shear

Velocity fields in turbulent flow can be explicitly simulated using computational fluid dynamics (CFD) or simplified analytical models ([Bowen et al. (2)](#_ENREF_2), [Taylor and Stocker (4)](#_ENREF_4)). Here a simplified approach is used (see discussion in main text).

*Generation of time-variable shear rate*

For the shear rate, we generally follow the approach of [Bowen et al. (2)](#_ENREF_2). We draw a shear rate (*E_b_*) from a log-normal distribution with mean (*E_b,ave_*, *E_bavg_* in ([2](#_ENREF_2))) and log variance (*E_b,logvar_*, *σ^2^* in ([2](#_ENREF_2))). We apply this shear rate for a duration of *f_shr_*/*E_b_* (5/*E_b_* in ([2](#_ENREF_2))). See Fig. S5B for a typical shear rate time series.

*Generation of velocity field*

The model includes a number of options for velocity fields. Options 1 and 2 are based on sinusoidal functions. Option 3 is a parabolic distribution.

For the sinusoidal fields, the period is set equal to the Kolmogorov length scale (estimated as *l_K_* = 0.25 (*ν* / *E_b_*)^0.5^), based on *E_b_* = 0.5 ( *ε* / *ν* )^0.5^, p. 40 in [Bowen et al. (2)](#_ENREF_2), and *l_K_* = ( *ν / ε* )^0.25^) subject to two constraints: (1) To adequately represent (i.e. sample) the distribution, the period needs to be at least twice the box size (2 *Δx*, i.e. Nyquist frequency). (2) To avoid abrupt changes in the velocity at the boundaries (e.g. across y = 0 for velocities in x direction), the model domain size needs to be divisible by the period. A random phase shift is used. The amplitude is set so that the average absolute gradient is equal to the shear rate (see below).

Option 1: Sinusoidal

This velocity field is based on a saturating sinusoidal function, see Fig. S5A for typical velocity fields. The direction of the velocity field is randomized at each interval. To avoid mass balance problems for the periodic boundary condition, the direction needs to align to one of the major axes in the model domain. To illustrate this constraint, consider a simple 2D model domain. If the velocity is along the x-axis and decreases with increasing *y*, then at each *y*, the flow rate leaving at the right equals that coming in at the left. If, however, the velocity is along a 45° angle with highest velocity at the bottom right corner, then the flow leaving at the right interface will generally be higher than that coming in at the left. The amplitude is calculated from the shear rate and period (*E_b_* = *v_shr,amp_* 4 / *v_shr,per_*).

Option 2: Random map procedure (RMP)

A velocity field was generated based on the “random map procedure” of [Young et al. (54)](#_ENREF_54) as modified for 3D by [Ehrlich et al. (55)](#_ENREF_55) (Fig. S8 in reference). See Movie S1 for an illustration of the velocity field. First, the advective displacement of (hypothetical) particles at the grid box centers over one (hypothetical) timestep (*dt_shr_*) are calculated using the following set of equations:

$$x\left( t+{dt}_{shr} \right)=x\left( t \right)+\frac{U_{shr}}{2} \left[ cos\left( 2 \pi\frac{y(t)}{v_{shr,per}}+\phi_{1} \right)+ cos\left( 2 \pi\frac{z(t)}{v_{shr,per}}+\phi_{2} \right) \right]$$

$$y\left( t+{dt}_{shr} \right)=y\left( t \right)+\frac{U_{shr}}{2} \left[ cos\left( 2 \pi\frac{x(t+{dt}_{shr})}{v_{shr,per}}+\omega_{1} \right)+cos\left( 2 \pi\frac{z(t)}{v_{shr,per}}+\omega_{2} \right) \right]$$

$$z\left( t+{dt}_{shr} \right)=z\left( t \right)+\frac{U_{shr}}{2} \left[ cos\left( 2 \pi\frac{x(t+{dt}_{shr})}{v_{shr,per}}+\theta_{1} \right)+cos\left( 2 \pi\frac{y(t+{dt}_{shr})}{v_{shr,per}}+\theta_{2} \right) \right]$$

where *U_shr_* (m) is the maximum displacement in one spatial dimension (*U* in ([55](#_ENREF_55))), *v_shr,per_* (m) is the period (*L* in ([55](#_ENREF_55))), and *φ_1_*, *φ_2_*, *ω_1_*, *ω_2_*, *θ_1_* and *θ_2_*, are independent random phases. Here, we use the displacement to calculate a velocity, vs. microbial displacement in ([54](#_ENREF_54), [55](#_ENREF_55)), and we therefore use a small max. displacement (*U_shr_* = *f_U,shr_* *Δxyz*, see Table S11). Then, the velocities are calculated based on the old and new positions (e.g. vel. in *x* dir. = ( *x*(*t*+*dt_shr_*) – *x*(*t*) ) / *dt_shr_* = ( *x*(*t*+*dt_shr_*) – *x*(*t*) ) *v_shr,amp_* / *U_shr_*, i.e. *dt_shr_* does not enter as a parameter). Then, velocities are interpolated from the center to the interfaces of the boxes. The amplitude is calculated from the shear rate and period (*E_b_* ≈ *v_shr,amp_* 2 / *v_shr,per_*), see Table S11).

Use of Option 1 and 2 in simulations

Both options are simplified methods that produce velocity fields with specified average shear rates, and it is not clear if either option is more realistic than the other. Option 2 produces more realistic looking velocity fields, but it includes three-dimensional velocities which are not compatible with the frameshift advection scheme (see Section S2.9.c), so it requires much smaller integration time steps and longer run times. Therefore, the faster Option 1 used in the full simulations.

Option 3: Parabolic

For testing, the model also supports a parabolic velocity profile (i.e. flow between two parallel plates, see Section S4.3, *v_shr_* = 2 *E_b_* [*y – y*^2^/(*ny* *Δy*)]).

*Numerical dispersion for chemicals*

For chemical advection, an explicit forward-time/backward-space (FTBS) integration scheme is used. This introduces numerical dispersion (a.k.a. false diffusion) in the direction of flow (*D_C,n_* = *v_shr_* *Δx* [0.5 – *v_shr_* *Δt* / ( 2 *Δx* ) ], Eqn. 12.19 in [Chapra (20)](#_ENREF_20)). For Option 1, with advection only one dimension, the average numerical dispersion in three dimensions is a third of that (*D_C,n_* / 3). The effective chemical diffusion in the model is the sum of the assigned diffusion coefficient (*D_C_*) and the numerical dispersion coefficient (*D_C_* + *D_C,n_*, see Section 11.6 in [Chapra (20)](#_ENREF_20)). The model computes *D_C,n_* for each time and location and subtracts it from *D_C_*, or assigns zero if *D_C_* < *D_C,n_*, which may happen at high shear rates.

1. Shear parameters

| **Parameter** | **Units** | **Value** | **Notes** |
| --- | --- | --- | --- |
| *E_b,ave_* | 1/s | 0.055 | = 0.005, thermocline or diel heating (a)  = 0.055 intermediate turbulence (c)  = 0.15, typical conditions in mixed layer (a)  = 0.5, surface, strong forcing (a)  = 0.5, relatively strong turbulence (c) |
| *E_b,logvar_* | - | 1.5 | = 1.5 (a) |
| *K_h,shr_* | - | 0.01 | Sinusoidal option  Shape factor (b) |
| *f_shr_* | - | 5.0 | = 5 (a) |
| *f_U,shr_* | - | 0.001 | RMP option  Affects mass balance error  = 0.001 corresponds to <0.1%/day (d) |

(a) [Bowen et al. (2)](#_ENREF_2).

(b) Smaller values produce a more abrupt shift, very large values produce a sine curve.

(c) [Taylor and Stocker (4)](#_ENREF_4), converted using Eqn. on p. 40 in [Bowen et al. (2)](#_ENREF_2).

(d) Based on tests with our model.

1. Shear: Illustration of method. (A) Typical velocity and shear profiles (shear rates indicated in panel B). (B) Typical shear rate (*E_b_*) time series.

### Sedimentation

Sedimentation is simulated using empirical relations between sedimentation velocity (*v_sed_*, m/s) and cell radius (*r*, m) (note units) for alive and dead cells (Fig. 3 in [Eppley et al. (56)](#_ENREF_56))

$$v_{sed}=c_{a}r^{c_{b}}$$

where the coefficients are: alive: *c_a_* = 1.40e1, *c_b_* = 1.26e0, dead: *c_a_* = 5.96e1, *c_b_* = 1.26e0. Sedimentation velocities corresponding to average cell sizes are listed in Table S12.

The wrapping boundary conditions in the model dictate that microbes settling out the bottom enter at the top. This does not agree with sedimentation being a loss process at a larger spatial scale. For the small model cube this means that (slightly) less cells should enter at the top than leave at the bottom. To account for this net loss, a removal rate based on the total water column depth (*k_sed_* = *v_sed_* / *H_sed_*, 1/day) is applied. This processes is implemented in a stochastic manner (see S2.8.g) and entails complete removal of selected microbes (incl. their attached bacteria).

1. Sedimentation parameters

| **Parameter** | **Units** | **Value (a)** | **Notes** |
| --- | --- | --- | --- |
| *v_sed,AVE_* | m/d | SPHY/alive: 0.0096  SPHY/dead: 0.041 | = 0.0028 (b)  = 0 (c)  = alive: 0.096, dead: 0.041 (d) |
|  |  | MPHY/alive: 0.049  MPHY/dead: 0.21 | = 0.038 (b)  = 0 (c)  = alive: 0.049, dead: 0.21 (d) |
|  |  | LPHY/alive: 0.10  LPHY/dead: 0.43 | = 0.12 (b)  = 0.40 (c)  = alive: 0.10, dead: 0.43 (d) |
| *H_sed_* | m | 26 | Average mixed-layer depth at BATS. |

(a) Value shown is for average cell size. Values in model vary individually.

(b) Based on Stokes’ Law, using 1.1 gWW/mL.

(c) Based on Fig. 2c in [Chindia and Figueredo (57)](#_ENREF_57), assuming spherical shape.

(d) Based on [Eppley et al. (56)](#_ENREF_56), see text.

### Chemotaxis and chemokinesis

The model used in the simulations uses run-reverse-flick chemotaxis, which is based on run & tumble chemotaxis. Here both submodels are described.

*Run & tumble model*

The run & tumble chemotaxis submodel was adopted from [Jackson (1)](#_ENREF_1). See Section S4.7 for a reproduction of one of the experiments in that reference. Bacteria move (i.e. run) at a specified constant velocity (*v_mot,ave_*, m/s) in a direction defined by their orientation (*θ*, *ϕ*, rad). Note that a Cartesian coordinate system is used here. During the run, the orientation changes based on a rotational diffusion coefficient (*D_r_*, rad^2^/d) (see Eqn. (10)).

The probability of a tumble (*P_t_*) in a timestep (*Δt*, d) is:

$$P_{t}=\frac{\Delta t}{\tau}$$

where *τ* (d) is the average run time, which is defined as:

$$\tau=\tau_{0} exp\left( \alpha\left[ \bar{\frac{dP_{b}}{dt}} \right] \right)$$

where *τ_0_* (d) is the average run time in the absence of a concentration gradient, *α* (d) is a constant that defines the increase of *τ* with $\left[ \bar{\frac{dP_{b}}{dt}} \right]$ (1/d), which is the running, exponentially decaying average of the rate of change of the fraction of bound surface receptors (*P_b_*). That value is updated each time step based on the present value:

$$\left[ \bar{\frac{dP_{b}}{dt}} \right]_{t+\Delta t}=\left( 1-\frac{\Delta t}{T_{m}} \right)\left[ \bar{\frac{dP_{b}}{dt}} \right]_{t}+\frac{\Delta t}{T_{m}}\left[ \frac{dP_{b}}{dt} \right]$$

where *T_m_* (d) is a time constant that defines the memory time scale. Higher values of *T_m_* correspond to longer memory. The rate of change of the fraction of bound surface receptors is:

$$\left[ \frac{dP_{b}}{dt} \right]=\frac{K_{D}}{\left( K_{D}+C \right)^{2}}\frac{\Delta C}{\Delta t}$$

where *K_D_* (mol/m^3^) is the half-saturation constant of the surface receptors and *C* (mol/m^3^) is the concentration. A run lasts for a minimum duration (*T_min_*, d). When a run stops (i.e. tumble), the bacteria orientation (*θ*, *ϕ*, rad.) is randomized.

*Run-reverse-flick & chemokinesis*

The run-reverse-flick and chemokinesis submodel is based on [Son et al. (5)](#_ENREF_5). See Fig. S19 for the reproduction of one of the experiments in that reference. The bacteria have a base speed in the absence of a chemoattractant (*v_i_*, m/s), which varies randomly among the individuals. This is assigned at division by drawing from a lognormal distribution with assigned mean (*v_mot,ave_*, m/s) and log variance (*v_logvar_*, m/s), truncated to avoid unrealistic values (*v_mot,min_*, *v_mot,max_*, m/s). The base speed is increased by a factor (*f_v_*) if the substrate concentration exceeds a threshold (*C_v_*, mol/m^3^).

The average run time is a function of the history of concentration experienced (as in the run & tumble model above) as well as the velocity. The relationship with the velocity is based on an empirical relationship between the reorientation frequency (*f(v)* (1/s) in ([5](#_ENREF_5)), 1/τ (s) here) and the velocity (*v_mot_*, µm/s) (Eq. S5 in ([5](#_ENREF_5))):

$$\tau_{v}(v_{mot})=\frac{c1}{1+e^{c2 \left( v_{mot}-c4 \right)}}+c3$$

where *c1* (*η* in ([5](#_ENREF_5)), s), *c2* (*ζ* in ([5](#_ENREF_5)), s/µm), *c3* (*θ* in ([5](#_ENREF_5)), s) and *c4* (*v_t_* in ([5](#_ENREF_5)), μm/s) are constants. The combined function is:

$$\tau=\tau_{0} exp\left( \alpha\left[ \bar{\frac{dP_{b}}{dt}} \right] \right)\frac{\tau_{v}(v_{mot})}{\tau_{v}(v_{mot,ave})}$$

The occurrence of a reorientation event is determined based on the above equation. When that happens, a forward-moving cell will reverse direction and start a backward run. A backward-moving cell will reverse direction and start moving forward and at that time may also flick, based on an empirical relationship between the probability of flicking and the velocity ([5](#_ENREF_5)):

$$P_{F}=c5+\frac{c6}{1+e^{c7 \left( v_{mot}-c8 \right)}}$$

where *c5*, *c6*, *c7* (s/µm) and *c8* (μm/s) are constants.

*Parameters*

The model equations and parameterization are generally based on the observations of [Son et al. (5)](#_ENREF_5). However, there is a discrepancy, because the model is three-dimensional, whereas the observations are in two dimensions. That means observed velocities do not include the third component. For a random orientation, the average ratio of 3D/2D velocities is 1.6 (based on our simulations), which was applied to the velocity parameters (e.g., *v_mot,ave_*, *c4*). Also, some flicks may appear as reversals or continuations in two dimensions. The actual frequency of reorientation and flicking are therefore also expected to be higher, but here the magnitude is more difficult to estimate as it also depends on the measurement precision and criteria for identifying these events. Corresponding parameters (e.g., *τ_0_*, *c5*) were therefore calibrated to match observations (see Fig. S19).

For simulations where cells with different transport characteristics (motility, chemotaxis, chemokinesis) are competed, the yield and respiration rate were adjusted to account for the metabolic cost of those processes (e.g. *ΔY_M_*, *k_R,C_*, see Section S3.6).

1. Chemotaxis parameters

| **Parameter** | **Units** | **Value** | **Notes** |
| --- | --- | --- | --- |
| *K_D_* | µmol/L | 0.03 | Function of chemical species (x)  = 100 (b)  = 3-1,000 (c)  = 10, 1-10 (e) |
| *τ_0_* | s | 0.3 (a1) | = 0.67 (b)  = 1.0 (c) |
| *T_m_* | s | 0.1 | = 1 (b)  = 0.6 (c)  = 0.1, 0.1-1 (e)  > *Δt* |
| *T_min_* | s | 0.0 | = 0 (b) (e)  = 0.2 (c) |
| *α* | s | 30 | = 660 (b) (c)  = 30 (e) |
| *D_r_* | rad^2^/s | 0.035 | = 0 (b)  = 0.035 (e)  = 0.062 (c) |
| *v_mot,ave_* | µm/s | 40 | = 12 (b)  = 12-80 (c)  = 40 (e) |
| *v_mot,logvar_* | - | 0.20 | = 0.20 (e) |
| *v_mot,min_* | µm/s | 5 | = 5 (e) |
| *v_mot,max_* | µm/s | 150 | = 150 (e) |
| *f_v_* | - | 1.3 | = 1.3 (e) |
| *C_v_* | µmol/L | 0.04 | Function of chemical species (x)  = 0.050 (e) |
| *c1* | s | -0.3942 | = -0.3942 (e) |
| *c2* | s/µm | -0.1285 (a2) | = -0.2019 (e) |
| *c3* | s | 0.8452 | = 0.8452 (e) |
| *c4* | µm/s | 29.66 (a2) | = 18.88 (e) |
| *c5* | - | 0.086 (a1) | = 0.055 (e) |
| *c6* | - | 1.1 (a1) | = 0.72 (e) |
| *c7* | s/µm | -0.16 (a2) | = -0.25 (e) |
| *c8* | µm/s | 57 (a2) | = 36 (e) |

(a1) Adjusted/calibrated to observations of [Son et al. (5)](#_ENREF_5), see text and Fig. S19.

(a2) Velocity parameters adjusted by 1.6 to account for unobserved third dimension (see text).

(b) [Jackson (1)](#_ENREF_1), Fig. 3 and Table 1.

(c) [Bowen et al. (2)](#_ENREF_2).

(e) [Son et al. (5)](#_ENREF_5). Velocity distribution parameters (*v_max_* etc.) based on fit to 3D/2D-corrected data in Fig. 4A of reference.

(x) Parameters corresponding to absolute concentrations (μmol/L) are expected to vary as a function of chemical species.


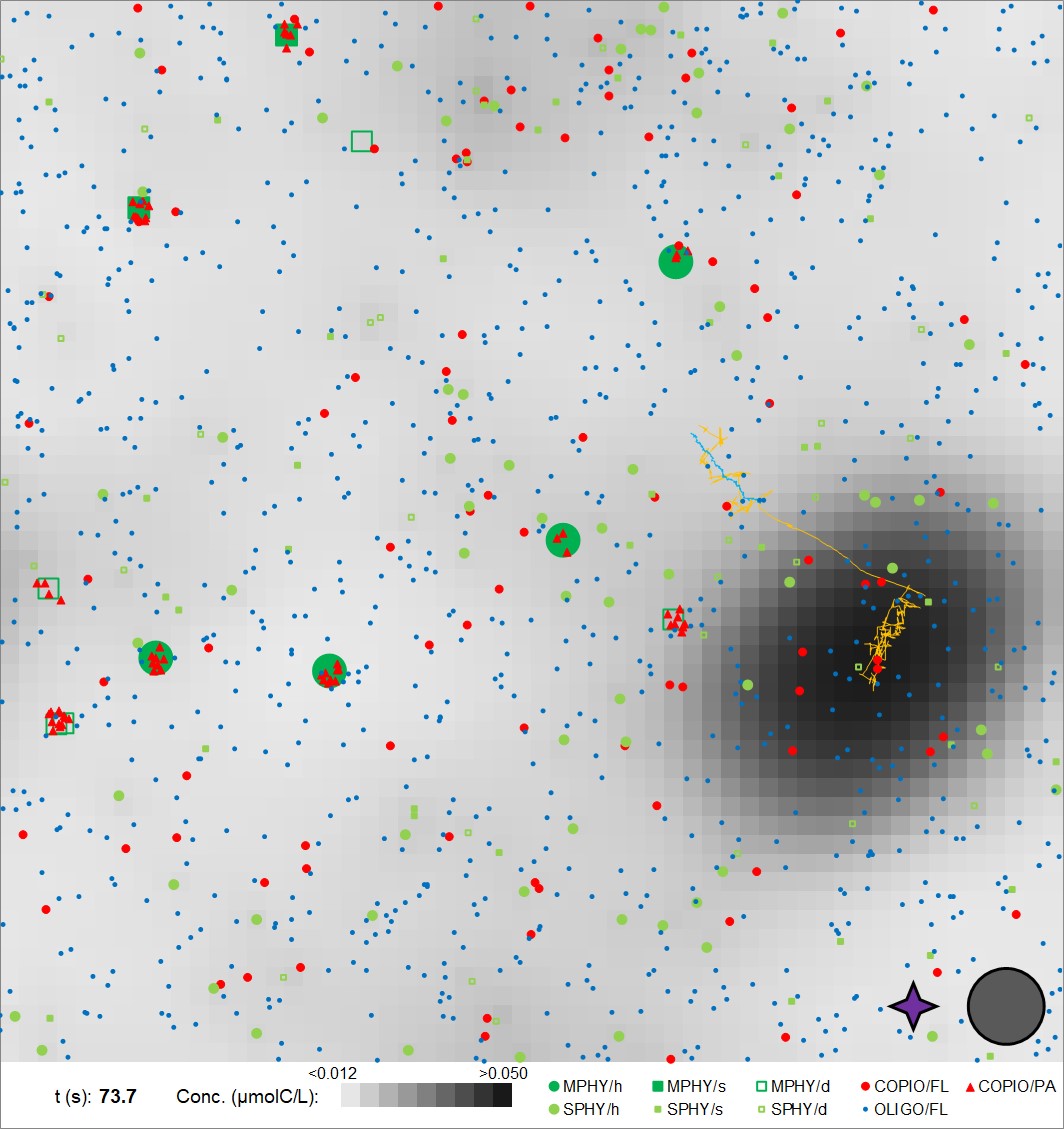


1. Chemotaxis: Illustration of method - map. See Fig. 1 caption. One copiotroph (COPIO, orange) and oligotroph (OLIGO, light) cells are traced. Traced COPIO also shown in Fig. S7. Note advection evident in OLIGO path. See Movie S2.

1. Chemotaxis: Illustration of method – time series. Relevant state and calculated variables of a selected copiotroph vs. time. (A) Concentration (*C*, μmol/L), previous concentration (*Cprev*, μmol/L), concentration gradient (*dCdt*, μmol/L/s), running, exponentially decaying average of the rate of change of the fraction of bound surface receptors (*adPbdt*, 1/s). (B) Average run time calculated (*tau*, s) and experienced (*tauX*, s). (C) Orientation (*theta*, *phi*, rad.) and change in orientation from previous time (*alpha*, rad.). (D) Position (*x*, *y*, *z*, μm) (relative to plot start time) and direction (*dv*). This cell is also shown in Fig. S6. Note that 2D view has dimension z on the vertical and y on the horizontal axes.

### Attachment

Copiotrophs attach to medium and large phytoplankters when they are within a specified distance (*a_dst_*, m) and the phytoplankter surface concentration (*C_S,P_*, mol/L) exceeds a threshold (*C_S,P,a_*, mol/L). The surface concentration is estimated based on the analytical solution to a continuous point source, using the production (exudation or carcass degradation) minus consumption (all attached bacteria) rates, and the phytoplankter radius:

$$C_{S,P}=\frac{W_{P}-W_{C}}{4 \pi D_{C} r} f_{C,S,P}$$

where *W_P_* and *W_C_* (molC/cell/s) are the production and consumption rates, respectively, and *r* (m) is the radius. A factor (*f_C,S,P_*) is included to account for non-uniform concentration and bacteria distribution (*f_C,S,P_* = 1 for uniform, *f_C,S,P_* > 1 for hotspots of concentration and bacteria).

Once attached they are transported with the phytoplankter. When the phytoplankter divides, the attached bacteria are randomly distributed among the daughter cells. When the phytoplankter is grazed, the bacteria are killed.

Attached bacteria consume substrate directly from the phytoplankter, i.e. not from the extracellular environment.

Bacteria detach based on a first-order detachment rate (*k_d_*), which decreases linearly with ambient concentration (for attached bacteria *C_S,P_*) below a detachment threshold concentration (*C_S,P,d_*, mol/L):

$$k_{d}=k_{d,max}-C_{S,P}\frac{k_{d,max}}{C_{S,P,d}}$$

Detachment is implemented as a stochastic process. A model that considers the effect of the substrate exposure history is presented by [Yawata et al. (58)](#_ENREF_58).

For simulations where cells with and without attachment are competed, the yield was adjusted to account for the metabolic cost of EPS production (*ΔY_A_*, see Section S3.6).

*Features not used in the simulations presented in the paper*

- Bacteria may detach at night.
- Detached bacteria may temporarily (*t_for_*) disable direction changes, e.g. tumbling, to “look” i.e. forage for another particle.
- Attached bacteria may increase exudation and speed up biomass loss and lysis (i.e. kill) senescent cells, as described in Section S2.8.c.

1. Attachment parameters

| **Name** | **Units** | **Value** | **Notes** |
| --- | --- | --- | --- |
| *f_C,S,P_* | - | 10 | - |
| *a_dst_* | μm | 0 | Touching |
| *k_d,max_* | 1/d | 100 | *k_d_* for *C_S,P_* = 0 |
| *C_S,P,a_* | μmol/L | 1.2 | > *C_S,P,d_* to avoid immediate detachment  > ~ (*k_L_* + *k_R_*) *K_S_* / *K_G,MAX,AVE_* to attach only if beneficial |
| *C_S,P,d_* | μmol/L | 0.0050 | Low enough to avoid detaching at night |
| *t_for_* | s | - | Not used. |
|  |  |  |  |

### Extracellular substrate

The mass balance for extracellular substrate, presented here for simplicity in one dimension (x) and positive velocity only, is:

$$\Delta x\Delta y\Delta z\frac{dC_{i}}{dt}=+v_{shr} \Delta y\Delta z C_{i-1}-v_{shr} \Delta y\Delta z C_{i}$$

$$+\frac{D_{C} \Delta y\Delta z}{\Delta x}\left( C_{i-1}-C_{i} \right)-\frac{D_{C} \Delta y\Delta z}{\Delta x}\left( C_{i}-C_{i+1} \right)+R$$

*R* (molC/d) accounts for interaction with microbes at that location and includes bacteria consumption (basic & regulation: Σ *k_G_* / *Y* *m*, storage: Σ *V* / *Y* *m*), phytoplankton exudation (Σ *k_m_* *A_cell_* *C_I,P_*) and phytoplankton death (initially: Σ *f_a_* *f_bst_ m* / *Δt*, then until *m* < *m_car,X_* Σ *k_m_* *A_cell_* *C_I,P_*, then Σ *f_a_* *m* / *Δt*, incl. dead cells only).

### Environment properties

The environment is a cube defined by the number of boxes (i.e. mass balance segments) in the x, y and z dimensions and their dimensions.

1. Environment properties

| **Name** | **Units** | **Value** | **Notes** |
| --- | --- | --- | --- |
| *nxyz* | - | 200 | - |
| *Δxyz* | µm | 50 | (a) |
| *V_env_* | mL | 1 | = (*nxyz*×*Δxyz*)^3^ |
|  |  |  |  |

(a) *Δx* = 50 μm corresponds to max. *D_C,n_* = 9.3e-11 for *v_shr,rel_* = 0.5 *Δx* / ( *Δt* × *xdtche* ) (see Sections S2.8.i and S2.9.c here, and Sections 11 and 12 in [Chapra (20)](#_ENREF_20)).

### Patch residence time calculation

A patch is defined by a threshold concentration (*pC_t_*, µmolC/L). A patch encounter starts when a cell first experiences a concentration above the threshold and stops when it first experiences a concentration below it. This time interval is the residence time inside the patch.

1. Patch definition

| **Name** | **Units** | **Value** | **Notes** |
| --- | --- | --- | --- |
| *pC_t_* | µmolC/L | 0.1 | = *C_R_*, see Table S5 |
|  |  |  |  |

## Implementation

### Platform

The model is implemented in FORTRAN 90 and OpenMP, with pre- and post-processing in MS Excel with Visual Basic for Applications (VBA). The source code is available from the corresponding author.

Parallelization involves adding up the production/consumption of each microbe within a model grid box for the corresponding term in the extracellular mass balance (*R* term in Eq. (24)), which is associated with round-off errors. The order in which microbes are processed can change in repeat simulations, due to different speeds of threads. This can lead to different round-off errors and consequently different results in repeat simulations (when multi-threading is used).

### Parallelization and batch stepping

The computational demand of the model is dominated by the chemical mass balance, i.e. the large number and small size of grid boxes, and small timestep required to maintain numerical stability. To allow for efficient utilization of parallel computing architecture, the environment was divided into “chunks” and each chunk was integrated independently for a number of time steps. During this time, chemical mass diffuses between the grid boxes within the chunk, but there is no exchange with neighboring chunks and the microbe source/sink term is constant. The scheme is illustrated in Fig. S8. The error introduced by this scheme can be controlled by limiting the number of chunks and time steps. Comparison BPD values (see main text) of the basecase to a simulation without chunk processing is presented in Fig. S9A.

1. Parallelization and batch stepping: Method illustration.

The model uses different time steps for different processes. The base time step is applied to the chemotaxis process, which requires the shortest time step. The time step for other processes, including microbe diffusion, microbe kinetics (e.g. growth, lysis) and the chemical mass balance use a time step larger by a specified factor. Comparison BPD values (see main text) of the basecase to a simulation without batch stepping is presented in Fig. S9B.

1. Parallelization and batch stepping: Testing. (A) Basecase vs. no chunk (CNK) processing. Difference in speed is ×5.5. Test run on full-scale basecase model, N = 1. (b) Basecase vs. no batch stepping (NDTBAT). Difference in speed is ×33. Test run on down-scaled (as in Movie S1) basecase model, N = 10.

### Frameshift advection

The model uses an explicit forward-time/backward-space (FTBS) integration scheme ([Chapra (20)](#_ENREF_20)). The highly simplified scenario in the model, including uniform geometry and constant shear velocities in one dimension allow for implementation using an algorithm that maintains numerical stability and limits numerical dispersion for very large velocities (i.e. *v_shr_* > *Δx* / ( *Δt* × *xdtche* )). Following is a description of this “frameshift” algorithm. The mass balance equation considering advection only is:

$$\Delta x\Delta y\Delta z\frac{dC_{i}}{dt}=+v_{shr} \Delta y\Delta z C_{i-1}-v_{shr} \Delta y\Delta z C_{i}$$

The numerical solution is:

$$\Delta x\Delta y\Delta z\frac{C_{i}^{t+\Delta t}-C_{i}^{t}}{\Delta t}=+v_{shr} \Delta y\Delta z C_{i-1}^{t}-v_{shr} \Delta y\Delta z C_{i}^{t}$$

$$C_{i}^{t+\Delta t}=C_{i}^{t}+\frac{v_{shr} \Delta t}{\Delta x} C_{i-1}^{t}-\frac{v_{shr} \Delta t}{\Delta x} C_{i}^{t}$$

Two example cases are presented in Fig. S10. The corresponding solutions are:

*v_shr_* = 0.5 × *Δx*: *v_shr,rel_* = 0.5 × *Δx* , *j* = *i*.

*v_shr_* = 2.5 × *Δx*: *v_shr,rel_* = 0.5 × *Δx* , *j* = *i* – 2.

$$C_{j}^{t+\Delta t}=C_{j}^{t}+\frac{v_{shr,rel} \Delta t}{\Delta x} C_{j-1}^{t}-\frac{v_{shr,rel} \Delta t}{\Delta x} C_{j}^{t}$$


1. Frameshift advection scheme.

### Attachment using spatial index

Attachment of bacteria to phytoplankters constitutes a form of direct agent-agent interaction which is computationally demanding, because for each bacteria, the distance to each phytoplankter has to be calculated (i.e. “loop in loop”) ([45](#_ENREF_45), [59](#_ENREF_59)). Here, this problem is solved using a spatial index. Specifically, we first populate an index, which gives the phytoplankter index present (if any) in each grid box. Then, for each bacterium, the closest phytoplankter is determined by searching those present in the 3×3×3 grid box vicinity. A limitation with this method is that it only considers one phytoplankter per grid box whereas more may be present. However, in our simulations that is rare and the error introduced not expected to be ecologically relevant.

1. Integration parameters

| **Name** | **Units** | **Value** | **Notes** |
| --- | --- | --- | --- |
| *Δt* | s | 0.067 | - |
| *ndtbat* | - | 200 | *ndtbat* < *V_box_* × *K_S_* × *Y* / ( *k_G,MAX_* × *m* × *Δt*) = 220 s (a) |
| *xdtche* | - | 50 | Controlled by numerical stability of the explicit integration scheme (b) |
| *xdtmD* | - | 200 | (b) |
| *xdtmDr* | - | 200 | (b) |
| *xdtmK* | - | 20 | *Δt* × *xdtmK* < *t_A_* (regulation activation time) |
|  |  |  |  |

(a) Limit corresponds to time it takes for one large (*m* = 2 × *m_0_*) stationary copiotroph to consume all substrate in one grid box. Calculation based on *C* << *K_S_*, *C* cancels out, time for higher concentrations is larger because of saturation. When there are more copiotrophs in one grid box they may consume more substrate than is present. In that case, the concentration may temporarily become negative, which conserves mass and does not affect the chemotaxis significantly (see Section S4.5).

(b) Valued used based on testing, see Section S4.

# Model parametrization

## Overview

An overview of model parameterization is presented in the main text. Specific parameter values incl. literature sources and notes are provided in the parameter tables in Section S2. This section presents additional discussion of model parameterization.

## Bacteria concentrations and cell sizes

### Bacteria concentrations

Target bacteria concentrations are based on observations. The cell concentrations for an individual bacteria species *i* (*X_c,i_*, cells/ml) (here just COPIO and OLIGO) is calculated from the total bacteria cell concentration (*X_c,T_*, cells/ml), gene copy fraction (*f_g,i_*, genes/genes) and factors for genes per genome (*α_i_*, genes/genome) and genome copies per cell (*β_i_*, genomes/cell). First, the total gene concentration (*X_g,T_*, genes/ml) is calculated:

$$X_{c,T}=\sum X_{c,i}=\sum\frac{X_{g,i}}{\alpha_{i} \beta_{i}}=\sum\frac{X_{g,T} f_{g,i}}{\alpha_{i} \beta_{i}}=X_{g,T}\sum\frac{f_{g,i}}{\alpha_{i} \beta_{i}}$$

$$X_{g,T}=\frac{X_{c,T}}{\sum\frac{f_{g,i}}{\alpha_{i} \beta_{i}}}$$

Then, the cell concentration for individual bacteria *i* is calculated:

$$X_{c,i}=\frac{X_{g,T} f_{g,i}}{\alpha_{i} \beta_{i}}$$

For classification into copiotrophic/oligotrophic bacteria, the data set “bats49month_rawdata_May_28_2021.csv”, containing 16S V1V2 amplicon sequence reads data from July 2016 till August 2020 with a monthly sampling interval, was assigned with the bats49month_taxonomy_phyloassigner_Nov_19_21.csv to the family level based on silva 123 database and PhyloAssigner reference tree ([60](#_ENREF_60)), and subsequently eukaryotes, chloroplasts, cyanobacteria and viruses removed. Each family was assigned as copiotrophic, oligotrophic or unassigned based on literature ([61-63](#_ENREF_61)). These data files are deposited under https://github.com/shutingliu/patch-model-16S-ASV.

For the factors specifying genome copies per cell (*β_i_*), a value of 1.5 was assumed for both COPIO and OLIGO (which actually cancels out in the analysis).

### Bacteria cell sizes

For the cell sizes, we assumed a COPIO/OLIGO cell size ratio of 5 and adjusted the OLIGO cell size to obtain an average cell size of 0.83e-15 molC/cell ([64](#_ENREF_64)).

### Copiotroph/oligotroph ratio (for diel cycles)

To test for diel patterns of copiotroph/oligotroph cell concentration ratios, we used two cruises with diel 16S V1V2 amplicon sequence sampling (~every 7-8 hours) from the BATS time-series: Cruise AE1819 from 2018/07/03 until 2018/07/06, and AE1916 from 2019/07/08 until 2019/07/12. We subsampled the ASV datasets of these cruises at the genus level for extreme oligotrophs and strong and extreme copiotrophs (see Fig. 2 in [Lauro et al. (61)](#_ENREF_61)), and calculated mean copiotrophic/oligotrophic ratios for the upper 50 m for each time-point and cruise. The appearing diel patterns were normalized for comparisons with the model outcomes (Fig. 2 main paper).

## Chemical diffusion coefficient

DOM is a mixture of chemicals with different diffusion coefficients, which are represented in the model as one chemical. The diffusion coefficient was set so that the oligotroph is not diffusion limited and can grow at the target growth rate at ambient concentrations (see Table S10). The copiotroph is mostly down regulated in the free-living phase, for which the growth rate is reduced and also below diffusion limitation.

## General basecase calibration

A number of input parameters for the basecase were calibrated to match target output parameters, as summarized in Table S18.

In addition, for copiotrophs, we adopt the following conceptual model and associated constraints on parameters:

- Growth is predominantly in the particle associated / attached phase, and the system is generally down-regulated in the free-living phase. That means the break-even and regulatory threshold concentrations need to be above the ambient concentration. See Section S3.5.
- Bacteria growth keeps up with phytoplankter growth. That sets a lower limit on the growth rate, which is controlled by *K_S_* and *k_G,MAX_* parameters.
- Bacteria do not spontaneously detach from (living) phytoplankters. That sets and upper limit on the detachment threshold (*C_S,P,d_*).

1. General basecase calibration

| **Input**  **Parameter** | **Output**  **Parameter** | **Units** | **Target** | **Notes** |
| --- | --- | --- | --- | --- |
| *Step 1* |  |  |  |  |
| *k_P,MAX_* | Net photosynthesis rate | 1/d | 0.50 | = photosynthesis rate – respiration rate.  Calculation based on biomass of all living cells.  See main text. |
| *k_sen_* | Fraction senescent | 1/d | 0.30 1/d | = senescent / alive cells  Basecase does not include senescent host killing.  Based on 5 – 60% in [Veldhuis et al. (65)](#_ENREF_65). |
| *k_K,b_* | Phytoplankton cell conc. | cells/mL | SPHY: 4.1e4  MPHY: 7.1e2 | Alive cells.  See main text. |
|  |  |  | LPHY: 1.0e2 |  |
| *Step 2* |  |  |  |  |
| *f_exp_, f_E_, k_E,b_* | Total bacteria C conc. | μmolC/L | 0.40 | *f_exp_* for MPHY & LPHY only, see Section S2.8.g.  *f_E_, k_E,b_* may change phytoplankton cell conc., Step 1  See main text. |
| COPIO *k_L_* | COPIO/OLIGO cell ratio  COPIO growth rate | -  1/d | 0.10  0.20 | *k_L_* up > *f_FL_* dn > *f_PA_* up > *k_G_* up  ([23](#_ENREF_23)) |
| OLIGO *k_L_*, *k_R_* | OLIGO growth rate | 1/d | 0.10 | ([23](#_ENREF_23)) |

## Regulation threshold concentration (*C_R_*)

Under steady conditions, there is a threshold concentration below which it is beneficial to turn the system off and *vice versa* (Fig. S11, referred to as steady-fast optimal regulatory concentration, *C_R,s,f_*). In a constant environment or for a regulation system that is fast, this would be the optimal regulatory concentration. In a variable environment and for a slower regulation system, there will be false on and false off cases, where the system has not responded to the new concentration. Here, the shapes of the on and off net growth rate functions suggest that a false off is worse than a false on. The optimal regulatory value is therefore expected to lie below that. How far will depend on the how fast the concentration experienced changes, which will be different for the two bacteria types and may vary across the concentration spectrum, in relation to the response time of the regulation system. If this concentration drops below the range of concentration experienced, then regulation is not beneficial.

For the copiotroph and the transcriptional regulation system, comparison of *C_R,s,f_* with the experienced concentration (Fig. S29) suggests that regulation is beneficial with a *C_R_* < ~0.1 μmol/L. For the oligotroph, *C_R,s,f_* generally lies below the experienced concentration suggesting regulation is not beneficial.

1. Regulation: Analysis of cost and benefit of regulation. (A) (B) Growth rate, respiration rate, lysis rate and net rate (*k_N_*) for copiotroph (COPIO) and oligotroph (OLIGO) and post-translational (S) and transcriptional (L) regulation systems over a range of concentrations. Analysis does not consider diffusion-limitation, see Fig. S3.

## Cost of motility, chemotaxis, chemokinesis and attachment

The various transport traits affect the respiration rate and yield. In the basecase simulation, those costs are implicitly included in the respiration rate and yield parameters, i.e. they are not separately quantified. For competition experiments with those features turned off (see main text), those costs are quantified and the corresponding parameters adjusted.

For the respiration rate, we first estimate the total energy consumption, in terms of organic carbon, as the consumption minus growth rates (Table S19). The cost of motility (m), chemotaxis (c) and chemokinesis (k) is a fraction of that, which is based on values for chemotaxis in *E. coli*, adjust upward for marine bacteria. Then, the relative rates of motility (propulsion), chemotaxis (sensing) and chemokinesis (sensing) are based on estimates for marine bacteria ([66](#_ENREF_66)), where we assume the cost for chemokinesis sensing is equal to that of chemotactic sensing. Note that, for the strain without chemokinesis, we adjust the average velocity parameter (*v_mot,ave_*) so that the average velocity is the same as for the strain with chemokinesis, so that there are no differences in propulsion cost. For example, a strain with motility, but without chemotaxis and chemokinesis, has a respiration rate equal to *k_R_* – *k_R,c_* – *k_R,k_*.

For the yield, the cost of motility, chemotaxis and chemokinesis is based on values for chemotaxis in *E. coli*, and the relative yields for motility, chemotactic sensing and chemokinesis sensing are based on estimates for marine bacteria ([66](#_ENREF_66)), where we again assume the cost for chemokinesis sensing is equal to that of chemotactic sensing. For example, a strain with motility, but without chemotaxis and chemokinesis, has a yield equal to *Y* + *ΔY_c_* + *ΔY_k_*.

For attachment, the yield of the non-attaching strain is increased to account for the metabolic cost of attachment (*ΔY_a_*). Previous models of bacterial biofilms have included costs of synthesis of extracellular polymeric substances (EPS) ([67](#_ENREF_67), [68](#_ENREF_68)), but costs are presumably not directly comparable with those of attachment to phytoplankters. For example, a strain without attachment has a yield equal to *Y* + *ΔY_a_*.

1. Parameterization: Cost of motility, chemotaxis, chemokinesis and attachment

| **Parameter** | **Units** | **Value** | **Notes** |
| --- | --- | --- | --- |
| *k_G_* | 1/d | 0.14 | Basecase model output |
| *Y* | - | 0.20 | Table S4 |
| Total energy rate (*A*) | 1/d | 0.56 | = *k_G_* / *Y* – *k_G_* |
| m+c+k energy fraction (*B*) | - | 0.032 | 0.1% for *E. coli* ([69](#_ENREF_69))  ×32 for marine bacteria (1-2 orders of magnitude higher, ([66](#_ENREF_66))). |
| m+c+k energy rate (*k_R,mck_*) | 1/d | 0.018 | = *A* × *B* |
| m energy rate (*k_R,m_*) | 1/d | 0.017 | (a) |
| c energy rate (*k_R,c_*) | 1/d | 0.00053 | (a) |
| k energy rate (*k_R,k_*) | 1/d | 0.00053 | (a) |
| k^–^ *v_mot,ave_* | µm/s | 46 | To yield same ave. vel. as k^+^ strain |
| Down-regulated respiration (*k_R,OFF_*) | 1/d | 0.035 | = 2 × *k_R,mck_* |
| *r_A_* | - | 0.65 | = (*k_R,ON_* – *k_R,OFF_*) / *k_R,ON_* |
| m+c+k yield fraction (*C*) | - | 0.02 | Fraction of total protein synthesis: 2% ([69](#_ENREF_69))  Fraction of total protein synthesis and energy:  “up to several percent” ([70](#_ENREF_70)) |
| m+c+k yield (*ΔY_mck_*) | - | 0.004 | = *Y* × *C* |
| m yield (*ΔY_m_*) | - | 0.0018 | (a) |
| c yield (*ΔY_c_*) | - | 0.0011 | (a) |
| k yield (*ΔY_k_*) | - | 0.0011 | (a) |
| Attachment yield fraction (*D*) | - | 0.10 | Assumed |
| Fraction attached (*E*) | - | 0.23 | Basecase model output |
| Attachment yield (*ΔY_a_*) | - | 0.0046 | = *Y* × *D* × *E* |

(a) Distribution based on Fig. 2 of ([66](#_ENREF_66)). Respiration rate: ATP/s, motility (propulsion) = 10^5^ (10^4^-10^6^), chemotactic sensing = 3.2×10^3^ (10^3^-10^4^), chemokinesis sensing = chemotactic sensing (assumed). Yield: proteins, motility (flagella) = 60,000, chemotactic sensing (chemosensory arrays + readout molecules) = 36,000 (20,000-30,000 + 11,000), chemokinesis sensing = chemotactic sensing (assumed).

# Model testing

A number of tests were performed to confirm the model was implemented correctly (i.e. no coding errors) and that the parallelization scheme does not introduce excessive error. Simplified scenarios where simulated and the results of the model were compared to analytical solutions or simple calculations (referred to as theoretical, THEO) or other models (e.g. [Jackson (1)](#_ENREF_1)). For full-scale scenarios with realistic parameters, both the un-parallelized (referred to as exact, EXA) and parallelized (referred to as approximation, APP) are presented. The overall disagreement between the EXA and APP simulations is less than 5%.

## Mass conservation

The model was run with an initial concentration and microbe population, and fixed, radiating or periodic boundary conditions, and the concentration after a long time was compared to the theoretical estimate (mass / total volume). These tests all confirm that the model conserves mass. The results are not presented here.

## DOM and microbe diffusion of an instantaneous release

This test includes simulation of chemical and microbe from an instantaneous point source. The theoretical concentration is available:

$$C\left( x,y,z,t \right)=\frac{M}{\left( 4 \pi D_{C} t \right)^{3/2}} exp\left( -\frac{x^{2}+y^{2}+z^{2}}{4 D_{C} t} \right)+C_{\infty}$$

where *C* (molC/m^3^) is the DOM concentration, *M* (molC) is the patch mass and *D_C_* (m^2^/s) is the DOM diffusion coefficient. *C_∞_* is the background concentration, which is assumed negligible here. The results are shown in Fig. S12.

1. Model testing: Diffusion from an instantaneous source. (A) DOM concentration time series at offset location (*x* = –250 µm). (B) DOM concentration transect through source location at 60 min. (C) Fraction of DOM mass and microbes remaining in a zone (*x*, *y*, *z* +/- 110 µm around source location). Parameters: *M* = 1.3e-10 molC, *D_C_* = 1e-10 m^2^/s.

## DOM and microbe diffusion of an instantaneous release with shear

In this case, the dispersion of an instantaneous planar source is simulated. Flow is in the x-direction and the velocity varies following a parabolic distribution in the y-direction. A range of shear rates are simulated, including low (L, *E_b_* = 0.05 1/s), medium (M, *E_b_* = 0.15 1/s) and high (H, *E_b_* = 0.30 1/s). The theoretical longitudinal dispersion coefficient (*D_disp_*, *k* in [Taylor (71)](#_ENREF_71), *D_T_* in [Leconte et al. (72)](#_ENREF_72)) that represents the combined effect of shear and molecular diffusion for this scenario is:

$$D_{disp}=\frac{\bar{U}^{2} b^{2}}{210 D_{diff}}$$

where $\bar{U}$ (m/s) is the average velocity (= *E_b_* *b* / 3), *b* (m) is the distance between the plates and *D_diff_* (m^2^/s) is the molecular diffusion coefficient. Note that *D_diff_* is different for chemical (= *D_C_*) and microbes (= *D_M_*), and for motile microbes it includes the random walk component (= *D_M_* + *D_mot_*, *D_mot_* = *v_mot_*^2^ *τ* / 3, ([52](#_ENREF_52))). The dispersion coefficient (*D_disp_*) is added to the molecular diffusion coefficient (*D_C_* or *D_M_*) for the dimension corresponding to the velocity direction. For the theoretical solution, the numerical dispersion was added in (see Section S2.8.i and [Chapra (20)](#_ENREF_20)). For the copiotroph, chemotaxis was turned off for this test (*α* = 0).

The results are shown in Fig. S13. Slight differences in net advection between the theoretical and EXA solution can be expected due to discretization of the velocity profile on the relatively coarse segmentation (*Δy* = 25 μm). Comparison of EXA and APP:

- L scenario: The APP simulation spreads the mass out less. This can be attributed to the smaller number of time steps used and resulting lower numerical dispersion.
- M scenario: The APP simulation plume is shifted left. This is due to the lower number of time steps and consequently lower output steps (i.e. the APP simulation cannot output results at exactly the same time as the EXA simulation).
- H scenario: The APP simulation is “wavy”, where each wave represents different rows of boxes that advect at different speeds and have not dispersed in the y-direction, again a result of the very low number of timesteps used (= 7 in the APP H simulation).

For copiotrophs, the spreading is slightly less, which can be attributed to the lack of numerical dispersion in the Lagrangian scheme. The dispersion for oligotrophs is much larger due to their low molecular diffusion coefficient (see equation above), and the peak is due to the center region that has relatively fast and uniform velocities (see panel A), which hasn’t mixed laterally.

1. Model testing: Diffusion from an instantaneous source with shear. (A) Velocity profiles. (B&C) Concentration and microbe profiles, at t = 2,000 μm / $\bar{\boldsymbol{U}}$. Profiles in panel C are for case M only. Theoretical distributions based on Eq. 10.19 in [Chapra (20)](#_ENREF_20). *Δx,y,z* = 25 μm, *D_C_* = 1e-11 m^2^/s.

## DOM diffusion from and microbe chemotaxis towards a continuous source

The steady-state concentration gradient for the continuous source is ([1](#_ENREF_1)):

$$C\left( x,y,z \right)=\frac{\left( \frac{W}{4 \pi D_{C}} \right)}{\sqrt{x^{2}+y^{2}+z^{2}}}+C_{\infty}$$

where *W* (molC/s) is the source rate. The results are presented in Fig. S14. The numerical solution drops off faster because it uses a zero-boundary condition at 0 and 2,000 µm, whereas the boundary for the analytical solution is at an infinite distance.

1. Model testing: Concentration from a continuous source. (A) DOM concentration transect through the source. (B) Average distance from source for copiotroph (motile) and oligotroph (non-motile) cells over time. Parameters: *W* = 3.0e-16 molC/s, *D_C_* = 1e-10 m^2^/s.

## DOM diffusion from and microbe chemotaxis towards a continuous source with consumption

This test includes a continuous source parameterized based on exudation from a large phytoplankter and ~10 chemotactic copiotrophs that consume the substrate. The results are presented in Fig. S15. The theoretical estimate of the concentration does not consider consumption (Eq. (33)) and has a boundary that is farther away, so it is generally higher.

1. Model testing: DOM diffusion from and microbe chemotaxis towards a continuous source with consumption. (A) DOM concentration vs. distance from source. *THEO does not consider consumption. (B) Bacteria concentration vs. distance from source. Steady-state distributions. Parameters: *W* = 3.0e-16 molC/s, *D_C_* = 1e-10 m^2^/s.

## High-resolution chemical profile

For chemicals, the model uses a relatively coarse grid (50 µm) and subsequently interpolates concentrations to a smooth surface. To evaluate ability of the model to simulate realistic patterns we simulate the chemical concentration resulting from 500 continuous sources or sinks. In this 8 µL volume, this corresponds to a concentration of 6.3e4 no./mL. The sources/sinks are distributed randomly throughout the environment, and the concentration profile along a random transect is compared to the analytical solution. For the analytical solution, the total concentration is the sum of the concentration from all source/sinks (Eq. (33)), using the principle of superposition ([20](#_ENREF_20)). The magnitude of the concentration in the model is different than that of the analytical solution. This can be explained by differences in boundary conditions. Specifically, for this simulation, the model uses open boundaries, which means the chemical mass is gained/lost by diffusion across the boundary. The analytical solution extends to infinity, so the concentration at the boundary is different.

1. Model testing: High-resolution chemical profile. (A) Concentration along a random transect in an environment including 500 random sources/sinks. Parameters: *W* = –5 to +5 e-16 molC/s, *D_C_* = 1e-10 m^2^/s, *C_∞_* = 100 µmolC/L.

## Chemotaxis towards a continuous source

This test includes reproducing the experiment presented in Fig. 3 of [Jackson (1)](#_ENREF_1), using run & tumble chemotaxis. The source is calculated as:

$$W=\frac{b a^{2.28} \mu f}{n}$$

where *b* (= 1.7e-4 molC/cm^2.28^, all parameter values from ([1](#_ENREF_1)), Fig. 3 and Table 1) is the carbon density, *a* (= 10 µm) is the radius, *µ* (= 1 /d) is the growth rate, *f* (= 1) is the organic matter leakage fraction of the phytoplankton cell and *n* (= 1) is the number of carbons per organic matter molecule. The results are presented in Fig. S17. The cells in this model exhibit a drift away from the source location, which is evident in the simulation with zero source and comparison to the Jackson model. In this model, this is due to the handling of the boundary conditions. Specifically, when cells reach the boundary they initiate a tumble, which has the effect of making them stay (i.e. “stick”) at the boundary longer than if they were reflected away. Since the outer boundary is much larger than the inner boundary (the alga), the net effect is a drift away from the center. This is consistent with how the boundaries is handled in the Jackson model, and it is not clear why that model does not exhibit this drift.

1. Model testing: Chemotaxis towards a continuous source. (A) Average distance from source. “J” corresponds to Fig. 3 of ([1](#_ENREF_1)). “M” corresponds to this model. Small differences between our model and that of Jackson can be attributed to stochasticity and difference concentration profile (numerical vs. analytical solution, see panel B). (B) DOM concentration profile through source. Parameters: as in Jackson paper.

## Chemotaxis towards a continuous source with shear

This test includes reproducing the experiment presented in Fig. 3 of [Bowen et al. (2)](#_ENREF_2), using run & tumble chemotaxis and shear. There are a number of differences between these two models:

- We use a square environment (vs. sphere).
- We use periodic boundaries (vs. radiating).
- To avoid buildup of chemical mass we include chemical decay (*K_C_* = 0.1/d).
- We use a sinusoidally-varying velocity field (see Section S2.8.i), whereas of Bowen et al. uses an analytical solution for flow around a sphere.
- The simulation includes a random shear rate time series, which is different from that used in the Bowen et al.
- The absolute source rate used is not clear to us from the information presented in the reference and our value is also not directly comparable to that (different boundary conditions and decay), and we use 6.5e-15 mol/s.

For these reasons, the results are expected to vary from those presented by [Bowen et al. (2)](#_ENREF_2). However, the general pattern of clustering at lower shear rates and *vice versa*, is consistent.

1. Model testing: Chemotaxis towards a continuous source with shear. (A) Fraction clustered. (B) Shear rate. Parameters: as in Bowen et al. paper.

## Chemotaxis along a linear gradient

This test includes reproducing the experiment presented in Fig. 2C (Inset 1) of [Son et al. (5)](#_ENREF_5).

1. Model testing: Chemotaxis along a linear gradient. Normalized concentration profile of bacteria in slow and fast speed bins.

## Bacteria and phytoplankton growth kinetics

Bacteria and phytoplankton were simulated in a batch reactor. The results are presented in Fig. S20. Similar tests were done to check exudation and lysis functions (results not shown).

1. Model testing: bacteria and phytoplankton growth kinetics. (A) Bacteria and DOM. (B) Phytoplankton and DIN. Parameters: Bacteria: *k_G,MAX_* = 1/d, *K_S_* = 1 µmolC/L, *Y* = 0.5, Phytoplankton: *k_P,MAX_* = 2/d, *K_S_* = 0.15 µmolN/L, *k_R_* = 0.2/d.

## Bacteria with and without storage

Bacteria with and without storage were simulated in a large batch reactor with low bacteria concentration (i.e. constant substrate concentration). The theoretical relationship between substrate concentration and growth rate for the storage model was obtained by solving the equations in Section S2.8.d for various growth rates (i.e. *k_G_* > *q* > *V* > *C*). The results are presented in Fig. S21.

1. Model testing: bacteria with (S) and without (NS) storage. (A) Net growth rate (*k_G_* – *k_R_*) vs. external substrate concentration. (B) Uptake rate and internal stored substrate vs. external substrate concentration.

## Phytoplankton and bacteria cultures

The model was used to simulate the culture laboratory experiments of [Becker et al. (73)](#_ENREF_73), [Paul et al. (74)](#_ENREF_74), [Wang et al. (75)](#_ENREF_75), [Moore et al. (76)](#_ENREF_76) and [Liu et al. (63)](#_ENREF_63). Those experiments were done with microbial species from the surface ocean, and ideally the model would be applied without any adjustments of parameters, i.e. a validation. However, the environmental conditions and microbial species in the basecase model do not correspond directly to those in these experiments. For example, several experiments were done with *Prochlorococcus* ecotypes (e.g. MED4), but the model species SPHY includes all cyanobacteria. Also, the model species OLIGO includes all oligotrophs. Further, there are several patterns in the observations that are unlikely to be representative of the ambient ocean, i.e. bottle effects. For example, even the unamended control experiment of [Liu et al. (63)](#_ENREF_63) shows large changes in the bacterial community over a few days. Additional examples are discussed below. Therefore, several parameters were re-calibrated for these applications (see Table S20 and Table S21).

The observations of [Becker et al. (73)](#_ENREF_73) show a beneficial effect of *Prochlorococcus* on SAR11 during the exponential phase of phytoplankton growth (Fig. S22). The model reproduces this pattern due to the exudation of DOM. The observations further show an antagonistic effect of *Prochlorococcus* on SAR11 when the phytoplankton enters stationary phase. The model does not include such a mechanism and therefore does not reproduce this pattern. However, it is questionable if this mechanism is relevant to our field simulation, because mass entering of phytoplankton cells into the stationary phase is attributed to batch culture experiments or large phytoplankton bloom events that do not occur at BATS. The observations also show a beneficial effect on three copiotrophs, where the effect is stronger when the phytoplankton enter the stationary phase (Fig. S23). The model is able to reproduce this pattern.

The data of [Paul et al. (74)](#_ENREF_74) show that *Thalassiosira pseudonana* supports the growth of *Dinoroseobacter shibae* in media without added organic carbon, which is reproduced by the model (Fig. S24). The experiments of [Wang et al. (75)](#_ENREF_75) with *Prorocentrum minimum* and *Dinoroseobacter shibae* also show this, although here the bacteria have an antagonistic effect on the phytoplankton when the later enter the stationary phase (Fig. S25). The model does not include an effect of bacteria on phytoplankton and that pattern is therefore not reproduced. It is unclear if this mechanism is relevant in the field, i.e. cultures with multiple bacteria show that other strains can protect the phytoplankton ([77](#_ENREF_77)). The observations of [Moore et al. (76)](#_ENREF_76) with *Thalassiosira pseudonana* and *Pelagibacter* sp. HTCC1062 (SAR11) show a positive effect of phytoplankton on bacteria during the exponential phase, which is reproduced by the model (Fig. S26).

[Liu et al. (63)](#_ENREF_63) performed experiments with the natural/ambient BATS surface ocean community under control (no added substrate, “CTRL”) and phytoplankton lysate derive substrate addition (“ADD”) (Fig. S27). The control experiment does not show a substantial increase in total bacteria concentration, but a shift towards copiotrophs. The increase in total bacteria concentration and shift towards copiotrophs is stronger in the addition experiment. The model reproduces these patterns.

1. Model testing: phytoplankton and bacteria cultures – *Prochlorococcus* and SAR11. Various strains of *Prochlorococcus* (see panel labels) and SAR11 (HTCC7211) in batch culture. Mono- (M) or co- (C) culture / phytoplankton (P) or bacteria (B) / data (d) or model (m). Model results for phytoplankton mono- and co-cultures are very close and may not be distinguishable. Data are from [Becker et al. (73)](#_ENREF_73).

1. Model testing: phytoplankton and bacteria cultures – *Prochlorococcus* and copiotrophs. Various strains of *Prochlorococcus* and copiotrophs (see panel labels) in batch culture. See legend Fig. S22.

1. Model testing: phytoplankton and bacteria co-culture – *Thalassiosira pseudonana* and *Dinoroseobacter shibae*. See legend Fig. S22. Data are from [Paul et al. (74)](#_ENREF_74).

1. Model testing: phytoplankton and bacteria cultures – *Prorocentrum minimum* and *Dinoroseobacter shibae*. See legend Fig. S22. *Bacteria concentration /100. Data are from [Wang et al. (75)](#_ENREF_75). Data for phytoplankton mono-culture are from the B_12_-replete experiment.

1. Model testing: phytoplankton and bacteria cultures – *Thalassiosira pseudonana* and *Pelagibacter* sp. HTCC1062 (SAR11). See legend Fig. S22. Data are from [Moore et al. (76)](#_ENREF_76).

1. Model testing: phytoplankton and bacteria cultures – Ambient field population. See legend Fig. S22. CTRL and ADD correspond to “S Control” and “S TW lysatePPL” in reference. *Model DOM includes constant recalcitrant background value (DOMr) based on min. observed concentration. Data are from [Liu et al. (63)](#_ENREF_63).
2. Phytoplankton and bacteria culture parameters (phytoplankton) (a)

| **Name** | **Units** | **MED4** | **MIT**  **9312** | **MIT**  **0801** | **MIT**  **9313** | **MIT**  **1314** | **MIT**  **1327** | ***T. pseud.***  **(f)** | ***P. min.*** | **Field**  **(g)** | **Notes** |
| --- | --- | --- | --- | --- | --- | --- | --- | --- | --- | --- | --- |
| *k_P,MAX_* | 1/d | 0.85 | 0.95 | 0.90 | 1.5 | 1.0 | 1.1 | 3.5, 1.8 | 0.75 | 0 | Table S3 |
| *f_E_* | - | 0.050 | 0.025 | 0.10 | 0.50 | 0.010 | - | 0.50 | 0.05 | - | Table S3 |
| *k_E,b_* | 1/d | 0.017 | 0.007 | 0.007 | 0.007 | 0.010 | - | 0.033, 0.10 | - | 1.0 | Table S3 |
| *f_a_* | - | - | - | - | - | 0.30-1.0  (b) | 0.10-0.50  (b) | - | - | - | Table S3 |
| *f_E,s_* | - | 1.0 | 3.0 | - | 0.2 | - | - | 1.0, 5.0 | 0.4 | - |  |
| *m_AVE_* | molC  /cell | - (c) | 8.7e-15  (c) | 1.7e-14  (c) | 8.0e-15  (c) | 7.3e-15  (c) | 2.8e-14  (c) | 1.7e-12  (d) | 3.9e-11  (e) | - | Table S8 |
|  |  |  |  |  |  |  |  |  |  |  |  |

(a) Only parameters that were changed from the base case are shown.

(b) Varies by bacteria strain.

(c) Based on medium nutrient concentration and stationary phase cell concentration.

(d) Based on Passow et al. <https://demo.bco-dmo.org/dataset/828942>.

(e) Based on <https://ncma.bigelow.org/CCMP1329>.

(f) Values for [Paul et al. (74)](#_ENREF_74), [Moore et al. (76)](#_ENREF_76).

(g) Natural/ambient field population.

1. Phytoplankton and bacteria culture parameters (bacteria) (a)

| **Name** | **Units** | **SAR11**  **HTCC**  **7211** | **MIT**  **1351** | **MIT**  **1352** | **MIT**  **1353** | ***D. shib.*** | **SAR11**  **HTCC**  **1062** | **Field**  **COPIO**  **(g)** | **Field**  **OLIGO**  **(g)** | **Notes** |
| --- | --- | --- | --- | --- | --- | --- | --- | --- | --- | --- |
| *k_G,MAX,AVE_* | 1/d | 0.85 | - | - | - | - | 0.40 | - | - | Table S8 |
| *K_S_* | μmolC  /L | 17.5 | 200 | 300 | 300 | - | 40 | - | - | Table S8 |
| *Y* | - | - | - | - | - | - | - | 0.25-0.45 (e) | - | Table S4 |
| *m_AVE_* | molC  /cell | - | 9.0e-15 | 1.5e-15 | 1.2e-15 | - | 3.0e-16 | 1.5e-15 | - | Table S8 |
|  |  |  |  |  |  |  |  |  |  |  |

(a) See footnote (a) in Table S20.

(g) Natural/ambient field population.

(e) Varies by treatment/DOM type.

# Additional model results

## Coexistence of copiotroph and oligotroph

Theory suggests that, in a constant environment, the number of species (here 2 bacteria) cannot be greater than the number of resources (here 1 DOM) ([78-80](#_ENREF_78)). We therefore asked if the co-existence of the copiotroph and oligotroph in the model is stable. Conclusively determining long-term coexistence is difficult due to the stochastic nature of the model and high computational cost, which prohibits long simulations. We confirmed stable coexistence by first running the model until an apparent equilibrium is reached and then re-running the model with higher/lower initial concentrations for the oligotrophs and copiotrophs, respectively. Because the cell concentrations from both simulations approach the apparent equilibrium we conclude stable coexistence (see Fig. S28).

The coexistence of two bacteria species on one resource can be explained by the spatially- and temporally-variable environment, and it is consistent with previous experimental and theoretical work ([78-80](#_ENREF_78)). In this case, the existence of the copiotroph along with the oligotroph in this oligotrophic environment is due to the microscale spatial heterogeneity (vs. temporal variability, e.g. ([81](#_ENREF_81))) and chemotaxis/attachment that create a niche for the copiotrophs. A macroscale model would not support coexistence of a copiotroph and oligotroph (on one DOM species) rather the oligotrophs would outcompete copiotrophs in such an environment. Explicit consideration of the microscale is therefore important for modeling even across broad classifications of bacteria.

1. Coexistence of copiotroph (COPIO) and oligotroph (OLIGO) in a microscale model with heterogeneous resource distribution. Basecase simulation. The model was initialized with bacteria concentrations below/above the equilibrium value to test for coexistence.

## Distribution of experienced substrate and growth rates

The distribution of substrate concentration experienced by the oligotroph is the same as that of the concentration in the environment (Fig. S29A, blue and black lines overlie, note log scales). The copiotroph experiences higher substrate concentrations due to chemotaxis and attachment to phytoplankton. The distribution pattern is consistent with the surface/phycosphere concentration of medium and large phytoplankters. The substrate concentration experienced translates to the growth rate via the Monod function (panel A > B > C). Copiotrophs regulate their growth system, so there are different functions for down-regulated (thin red line in panel B) and up-regulated (heavy red line) modes. Most free-living copiotrophs are down-regulated, but ~1% experience higher concentrations near phytoplankters or patches (see Fig. 1 and Movie S1) and those are up-regulated and growing faster.

1. Distribution of DOM substrate and growth rates of copiotrophs and oligotrophs. Basecase simulation. (A) Distributions of extracellular concentration (Cext), concentration experienced by copiotrophs (COPIO), oligotrophs (OLIGO), and surface (i.e. phycosphere) substrate concentration of medium and large phytoplankters (MPHY, LPHY). (B) Monod plot of growth rate vs. substrate concentration. Substrate concentrations and growth rates unconventionally presented as log10 values to illustrate distribution over large range. (C) Distribution of growth rates. Cave = average substrate concentration experienced, kGave = average growth rate.

## Population structure

The cell size distribution for the oligotroph in the model deviates from the theoretical size distribution based on constant growth rate in two ways (Fig. S30D). First, it is more gradual, vs. step-like, at the lower end, which is due to imperfect biomass division (i.e. randomization of the biomass split fraction at division ([36](#_ENREF_36), [45](#_ENREF_45))). Second, the larger cells are slightly overrepresented, because the growth rate slows down with size, due to the decrease in the surface area : volume ratios, which slows their progression through the later part of the cell cycle.

The pattern for the copiotrophs is more complex and affected by the different phases, that include free-living and particle-associated (incl. to medium and large phytoplankters, each with healthy, senescent and dead lifecycle stages) and associated differences in experienced substrate concentration and growth rate (incl. up/down-regulation), and loss rate (i.e. co-grazing of attached cells), and interaction among the phases (i.e. attachment and detachment). Bacterial cells at the low end of the size spectrum are mostly free-living cells that experience very low substrate concentrations and growth rates, and have shrunk to this size. Because free-living cells are growing very slowly, they are over-represented in the cell size distribution. Larger bacterial cells are mostly attached to phytoplankters. These cells experience high substrate concentrations and growth rates, resulting in rapid progression though this part of the cell cycle causing under-representation in the cell size distribution.

1. Population structure of copiotroph (COPIO) and oligotroph (OLIGO) bacteria. Cell size is normalized to average size after division. (A) Growth rate. Growth rate normalized to average. THEO is theoretical distribution based on specific surface area (see SI). (B) Experienced substrate concentration. “trend” is linear fit. (C) Free-living fraction. (D) Cell size distribution. THEO is theoretical distribution for exact 50/50 biomass split at division and constant growth rate (i.e. no dependence on specific surface area) ([36](#_ENREF_36)).

## Chemotaxis towards phytoplankters

To quantify the effect of chemotaxis, we calculate the average distance from bacteria to the nearest phytoplankter (bacteria-phytoplankter distance, BPD, Table S22, see also main paper). Ideally, without chemotaxis or reproductive clustering, i.e. due to time required for daughter cells to disperse following division ([54](#_ENREF_54)), this should be equal to the average nearest neighbor distance of the phytoplankters (phytoplankter-phytoplankter distance, PPD). In the model, reproductive clustering reduces the PPD below that computed based on the cell concentration, especially for the larger phytoplankters. The difference increases when shear is removed (discussed further below) and disappears when phytoplankton offspring are placed (unrealistically) at random locations within the model domain (results not presented). For the oligotroph, the BPD to all phytoplankters is close to the PPD of the phytoplankters, but slightly higher for the larger phytoplankters, due to the reproductive clustering.

To prevent the clustering effect from affecting the analysis, we quantify the chemotactic efficiency of copiotroph as the difference between the oligotroph and copiotroph BPD (ΔBPD, Table S22, Fig. 3A). The ΔBPD to small phytoplankters is larger due to chemotaxis. The effect is much stronger for dead small phytoplankters cells (ΔBPD = 42 μm), but here we focus on the association with living cells. The ΔBPD to medium and large phytoplankters shows no effect of chemotaxis.

1. Selected distance metrics (μm). Basecase (values bold), no shear (values in [ ]) and reproductive clustering diagnostic (values in < >) simulations. Mean (SEM) (a).

|  | **PPD** | | | **BPD** | | |
| --- | --- | --- | --- | --- | --- | --- |
|  | **Model** | **Theo.** | **Model/Theo.** | **COPIO** | **OLIGO** | **OLIGO-COPIO**  **(ΔBPD)** |
| SPHY | **169 (<1)**  [166 (<1)]  <170 (<1) > | **170**  [171]  <170> | **0.99**  [0.98]  <1.00> | **161 (<1)**  [161 (<1)]  <161 (<1)> | **171 (<1)**  [173 (<1)]  <171 (<1)> | **10**  [12]  <10> |
| MPHY | **645 (1)**  [556 (1)]  <663 (1)> | **665**  [668]  <662> | **0.97**  [0.83]  <1.00> | **669 (<1)**  [691 (<1)]  <662 (<1)> | **670 (<1)**  [715 (<1)]  <663 (<1)> | **0**  [24]  <1> |
| LPHY | **1,251 (5)**  [967 (6)]  <1,296 (6)> | **1,273**  [1261]  <1,301> | **0.97**  [0.77]  <1.00> | **1,296 (<1)**  [1,313 (<1)]  <1,302 (<1)> | **1,295 (<1)**  [1,376 (<1)]  <1,301 (<1)> | **-1**  [62]  <-1> |

(a) All metrics for healthy phytoplankters, free-living bacteria. SPHY, MPHY, LPHY = small, medium, large phytoplankters, COPIO = copiotrophs, OLIGO = oligotrophs, PPD = phytoplankter-phytoplankter distance, BPD = bacteria-phytoplankter distance.

1. Distribution of substrate experienced by FL bacteria. See Fig. S29 legend.

## Fitness effect of transcriptional regulation (in the free-living phase)

Previous work suggests that chemotaxis is a pre-requisite to transcriptional regulation of nutrient assimilation systems, because it increases the patch residence time beyond the time required to activate the system, i.e. transcription, translation and protein maturation ([8](#_ENREF_8)). To explore this question further, we perform competition experiments with copiotrophic strains that regulate their metabolism differently. Note that, in the model, attached cells are always up-regulated, so this analysis only applies to free-living cells. Parameters for regulation are based on transcriptional (vs. post-translational) regulation (see Section S3). We adjust the regulatory threshold concentration, where zero corresponds to no regulation (i.e. the system is always on) and higher values correspond to more frequent down-regulation. The results suggest that, for the copiotroph, there is a strong benefit of regulation (Fig. S32). Note that the small benefit in the no-regulation case (COPIO/0) is due to random noise. By down-regulating the system when the substrate concentration is low, the growth rate decreases, but this is compensated for by lower respiration. For the oligotroph, there is no benefit of regulation.

Why is it beneficial for the copiotroph to regulate and not the oligotroph? One reason is that the patch residence time is short ([8](#_ENREF_8)). Using a patch definition equal to the regulation threshold, the residence time of the copiotroph and oligotroph are 61 and 1.6 min., respectively, which are above and below the activation time of 5 min. Another explanation is that the distribution of substrate concentrations experienced, which is wider for the copiotroph (due to chemotaxis) than for the oligotroph (**Error! Reference source not found.**). Attachment, which is not considered in these simulations will further increase the patch residence time of copiotrophs and the range of substrate experienced (Fig. S29 vs. **Error! Reference source not found.**).

1. Cost and benefit of transcriptional regulation. Copiotroph (COPIO) and oligotroph (OLIGO) with regulation (R) vs. without regulation (NR) for various regulatory threshold concentrations. * = basecase scenario (A) Fitness coefficient (*f*). (B) Fraction the system is active (fA). (C) Growth and respiration rates (kG, kR).

## Effect of increasing shear and sedimentation

1. Effect of increasing shear and sedimentation. See Fig. 5 legend.

# References

1. Jackson GA (1987) Simulating chemosensory responses of marine microorganisms1. *Limnology and Oceanography* 32(6):1253-1266.

2. Bowen JD, Stolzenbach KD, & Chisholm SW (1993) Simulating bacterial clustering around phytoplankton cells in a turbulent ocean. *Limnology and Oceanography* 38(1):36-51.

3. Blackburn N, Azam F, & Hagström Å (1997) Spatially explicit simulations of a microbial food web. *Limnology and Oceanography* 42(4):613-622.

4. Taylor JR & Stocker R (2012) Trade-Offs of Chemotactic Foraging in Turbulent Water. *Science* 338(6107):675-679.

5. Son K, Menolascina F, & Stocker R (2016) Speed-dependent chemotactic precision in marine bacteria. *Proceedings of the National Academy of Sciences* 113(31):8624-8629.

6. Brumley DR*, et al.* (2019) Bacteria push the limits of chemotactic precision to navigate dynamic chemical gradients. *Proceedings of the National Academy of Sciences* 116(22):10792-10797.

7. Christensen AK, Piggott MD, van Sebille E, van Reeuwijk M, & Pawar S (2022) Investigating microscale patchiness of motile microbes under turbulence in a simulated convective mixed layer. *PLOS Computational Biology* 18(7):e1010291.

8. Noell SE*, et al.* (2023) Differences in the regulatory strategies of marine oligotrophs and copiotrophs reflect differences in motility. *Environmental Microbiology* 25(7):1265-1280.

9. Raina J-B*, et al.* (2023) Chemotaxis increases metabolic exchanges between marine picophytoplankton and heterotrophic bacteria. *Nature Microbiology* 8(3):510-521.

10. Grimm V*, et al.* (2010) The ODD protocol: A review and first update. *Ecological Modelling* 221(23):2760-2768.

11. Di Toro DM (1980) Applicability of cellular equilibrium and monod theory to phytoplankton growth kinetics. *Ecological Modelling* 8:201-218.

12. Schnoor JL (1996) *Environmental modeling: fate and transport of pollutants in water, air, and soil* (John Wiley and Sons).

13. Morel FMM (1987) KINETICS OF NUTRIENT UPTAKE AND GROWTH IN PHYTOPLANKTON1. *Journal of Phycology* 23(1):137-150.

14. Weitz JS*, et al.* (2015) A multitrophic model to quantify the effects of marine viruses on microbial food webs and ecosystem processes. *The Isme Journal* 9:1352.

15. Hellweger FL & Lall U (2004) Modeling the Effect of Algal Dynamics on Arsenic Speciation in Lake Biwa. *Environmental Science & Technology* 38(24):6716-6723.

16. Painting SJ, Moloney CL, & Lucas MI (1993) Simulation and field measurements of phytoplankton-bacteria-zooplankton interactions in the southern Benguela upwelling region. *Marine Ecology Progress Series* 100(1/2):55-69.

17. Baines SB & Pace ML (1991) The production of dissolved organic matter by phytoplankton and its importance to bacteria: Patterns across marine and freshwater systems. *Limnology and Oceanography* 36(6):1078-1090.

18. Moran MA*, et al.* (2022) The Ocean's labile DOC supply chain. *Limnology and Oceanography* n/a(n/a).

19. Connolly JP, Coffin RB, & Landeck RE (1992) Modeling carbon utilization by bacteria in natural water systems. *Modeling the Metabolic and Physiologic Activities of Micro-organisms*, ed Hurst CJ (Wiley, New York), pp 249-276.

20. Chapra SC (1997) *Surface Water-Quality Modeling* (McGraw-Hill, Boston).

21. Mentges A, Feenders C, Deutsch C, Blasius B, & Dittmar T (2019) Long-term stability of marine dissolved organic carbon emerges from a neutral network of compounds and microbes. *Scientific Reports* 9(1):17780.

22. Bucci V, Hoover S, & Hellweger F (2011) Modeling Adaptive Mutation of Enteric Bacteria in Surface Water Using Agent-Based Methods. *Water Air Soil Pollut* 223(5):2035–2049.

23. Kirchman DL (2016) Growth Rates of Microbes in the Oceans. *Annual Review of Marine Science* 8(1):285-309.

24. Long AM, Hou S, Ignacio-Espinoza JC, & Fuhrman JA (2019) Benchmarking metagenomic marine microbial growth prediction from codon usage bias and peak-to-trough ratios. *bioRxiv*:786939.

25. Vieira-Silva S & Rocha EPC (2010) The Systemic Imprint of Growth and Its Uses in Ecological (Meta)Genomics. *PLOS Genetics* 6(1):e1000808.

26. Muscarella ME, Howey XM, & Lennon JT (Trait-based approach to bacterial growth efficiency. *Environmental Microbiology* n/a(n/a).

27. Giorgio PAd & Cole JJ (1998) BACTERIAL GROWTH EFFICIENCY IN NATURAL AQUATIC SYSTEMS. *Annual Review of Ecology and Systematics* 29(1):503-541.

28. Ihssen J & Egli T (2005) Global physiological analysis of carbon‐and energy‐limited growing Escherichia coli confirms a high degree of catabolic flexibility and preparedness for mixed substrate utilization. *Environmental microbiology* 7(10):1568-1581.

29. Egli T (2010) How to live at very low substrate concentration. *Water Research* 44(17):4826-4837.

30. Gómez-Consarnau L*, et al.* (2019) Microbial rhodopsins are major contributors to the solar energy captured in the sea. *Science Advances* 5(8):eaaw8855.

31. Walter JM, Greenfield D, Bustamante C, & Liphardt J (2007) Light-powering Escherichia coli with proteorhodopsin. *Proceedings of the National Academy of Sciences* 104(7):2408-2412.

32. Kiørboe T (2018) A mechanistic approach to plankton ecology. *A Mechanistic Approach to Plankton Ecology*, (Princeton University Press).

33. Hellweger FL (2018) Heterotrophic substrate specificity in the aquatic environment: The role of microscale patchiness investigated using modelling. *Environmental Microbiology* 20(10):3825-3835.

34. Hellweger FL, Kravchuk ES, Novotny V, & Gladyshev MI (2008) Agent-Based Modeling of the Complex Life Cycle of a Cyanobacterium (Anabaena) in a Shallow Reservoir. *Limnology and Oceanography* 53(4):1227-1241.

35. Thingstad TF (1987) Utilization of N, P, and organic C by heterotrophic bacteria. I. Outline of a chemostat theory with a consistent concept of 'maintenance' metabolism. *Marine Ecology Progress Series* 35(1/2):99-109.

36. Hellweger FL & Kianirad E (2007) Individual-based modeling of phytoplankton: Evaluating approaches for applying the cell quota model. *Journal of Theoretical Biology* 249(3):554-565.

37. Hellweger FL (2008) The role of inter-generation memory in diel phytoplankton division patterns. *Ecological Modelling* 212(3–4):382-396.

38. Jacquet S, Partensky F, Lennon J-F, & Vaulot D (2001) DIEL PATTERNS OF GROWTH AND DIVISION IN MARINE PICOPLANKTON IN CULTURE. *Journal of Phycology* 37(3):357-369.

39. Fredrick ND, Berges JA, Twining BS, Nuñez-Milland D, & Hellweger FL (2013) Exploring Mechanisms of Intracellular P Heterogeneity in Cultured Phytoplankton using Agent Based Modeling. *Applied and Environmental Microbiology*.

40. Kreft J-U, Booth G, & Wimpenny JWT (1998) BacSim, a simulator for individual-based modelling of bacterial colony growth. *Microbiology* 144(12):3275-3287.

41. Hellweger FL (2010) Resonating circadian clocks enhance fitness in cyanobacteria *in silico*. *Ecological Modelling* 221(12):1620-1629.

42. Li C*, et al.* (2022) Quasi-Antiphase Diel Patterns of Abundance and Cell Size/Biomass of Picophytoplankton in the Oligotrophic Ocean. *Geophysical Research Letters* 49(5):e2022GL097753.

43. DuRand MD, Olson RJ, & Chisholm SW (2001) Phytoplankton population dynamics at the Bermuda Atlantic Time-series station in the Sargasso Sea. *Deep Sea Research Part II: Topical Studies in Oceanography* 48(8):1983-2003.

44. Loferer-Krößbacher M, Klima J, & Psenner R (1998) Determination of Bacterial Cell Dry Mass by Transmission Electron Microscopy and Densitometric Image Analysis. *Applied and Environmental Microbiology* 64(2):688-694.

45. Hellweger FL & Bucci V (2009) A bunch of tiny individuals—Individual-based modeling for microbes. *Ecological Modelling* 220(1):8-22.

46. Hellweger FL, Jabbur ML, Johnson CH, van Sebille E, & Sasaki H (2019) Circadian clock helps cyanobacteria manage energy in coastal and high latitude ocean. *The ISME Journal*.

47. Shirani S & Hellweger FL (2017) Neutral Evolution and Dispersal Limitation Produce Biogeographic Patterns in Microcystis aeruginosa Populations of Lake Systems. *Microb Ecol*:1-11.

48. Rabouille S, Edwards CA, & Zehr JP (2007) Modelling the vertical distribution of Prochlorococcus and Synechococcus in the North Pacific Subtropical Ocean. *Environmental Microbiology* 9(10):2588-2602.

49. Mine AH, Coleman ML, & Colman AS (2021) Phosphorus Release and Regeneration Following Laboratory Lysis of Bacterial Cells. *Frontiers in Microbiology* 12.

50. Grossart H-P & Simon M (1998) Bacterial colonization and microbial decomposition of limnetic organic aggregates (lake snow). *Aquatic Microbial Ecology* 15(2):127-140.

51. Takeshi M & Norio Y (2005) Theoretical model of interactions between particle-associated and free-living bacteria to predict functional composition and succession in bacterial communities. *Aquatic Microbial Ecology* 39(1):35-46.

52. Lambert BS, Fernandez VI, & Stocker R (2019) Motility drives bacterial encounter with particles responsible for carbon export throughout the ocean. *Limnology and Oceanography Letters* 4(5):113-118.

53. Smriga S, Fernandez VI, Mitchell JG, & Stocker R (2016) Chemotaxis toward phytoplankton drives organic matter partitioning among marine bacteria. *Proceedings of the National Academy of Sciences* 113(6):1576-1581.

54. Young WR, Roberts AJ, & Stuhne G (2001) Reproductive pair correlations and the clustering of organisms. *Nature* 412(6844):328-331.

55. Ehrlich E, Thygesen UH, & Kiørboe T (2022) Evolution of toxins as a public good in phytoplankton. *Proceedings of the Royal Society B: Biological Sciences* 289(1977):20220393.

56. Eppley RW, Holmes RW, & Strickland JDH (1967) Sinking rates of marine phytoplankton measured with a fluorometer. *Journal of Experimental Marine Biology and Ecology* 1(2):191-208.

57. Chindia JA & Figueredo CC (2018) Phytoplankton settling depends on cell morphological traits, but what is the best predictor? *Hydrobiologia* 813(1):51-61.

58. Yawata Y, Carrara F, Menolascina F, & Stocker R (2020) Constrained optimal foraging by marine bacterioplankton on particulate organic matter. *Proceedings of the National Academy of Sciences* 117(41):25571-25579.

59. Kreft J-U, Picioreanu C, Wimpenny JWT, & van Loosdrecht MCM (2001) Individual-based modelling of biofilms. *Microbiology* 147.

60. Vergin KL, Done B, Carlson CA, & Giovannoni SJ (2013) Spatiotemporal distributions of rare bacterioplankton populations indicate adaptive strategies in the oligotrophic ocean. *Aquatic Microbial Ecology* 71(1):1-13.

61. Lauro FM*, et al.* (2009) The genomic basis of trophic strategy in marine bacteria. *Proceedings of the National Academy of Sciences* 106(37):15527-15533.

62. Yooseph S*, et al.* (2010) Genomic and functional adaptation in surface ocean planktonic prokaryotes. *Nature* 468(7320):60-66.

63. Liu S*, et al.* (2020) Stable Isotope Probing Identifies Bacterioplankton Lineages Capable of Utilizing Dissolved Organic Matter Across a Range of Bioavailability. *Frontiers in Microbiology* 11.

64. Steinberg DK*, et al.* (2001) Overview of the US JGOFS Bermuda Atlantic Time-series Study (BATS): a decade-scale look at ocean biology and biogeochemistry. *Deep Sea Research Part II: Topical Studies in Oceanography* 48(8):1405-1447.

65. Veldhuis M, Kraay G, & Timmermans K (2001) Cell death in phytoplankton: correlation between changes in membrane permeability, photosynthetic activity, pigmentation and growth. *European Journal of Phycology* 36(2):167-177.

66. Keegstra JM, Carrara F, & Stocker R (2022) The ecological roles of bacterial chemotaxis. *Nature Reviews Microbiology* 20(8):491-504.

67. Nadell CD, Xavier JB, Levin SA, & Foster KR (2008) The Evolution of Quorum Sensing in Bacterial Biofilms. *PLOS Biology* 6(1):e14.

68. Kreft J-U & Wimpenny JW (2001) Effect of EPS on biofilm structure and function as revealed by an individual-based model of biofilm growth. *Water Science and Technology* 43(6):135-135.

69. Neidhardt FC (1996) *Escherichia coli and Salmonella: cellular and molecular biology*.

70. Colin R, Ni B, Laganenka L, & Sourjik V (2021) Multiple functions of flagellar motility and chemotaxis in bacterial physiology. *FEMS Microbiology Reviews* 45(6).

71. Taylor GI (1953) Dispersion of soluble matter in solvent flowing slowly through a tube. *Proceedings of the Royal Society of London. Series A. Mathematical and Physical Sciences* 219(1137):186-203.

72. Leconte M, Martin J, Rakotomalala N, Salin D, & Yortsos YC (2004) Mixing and reaction fronts in laminar flows. *The Journal of Chemical Physics* 120(16):7314-7321.

73. Becker JW, Hogle SL, Rosendo K, & Chisholm SW (2019) Co-culture and biogeography of Prochlorococcus and SAR11. *The ISME Journal* 13(6):1506-1519.

74. Paul C, Mausz MA, & Pohnert G (2013) A co-culturing/metabolomics approach to investigate chemically mediated interactions of planktonic organisms reveals influence of bacteria on diatom metabolism. *Metabolomics* 9(2):349-359.

75. Wang H, Tomasch J, Jarek M, & Wagner-Döbler I (2014) A dual-species co-cultivation system to study the interactions between Roseobacters and dinoflagellates. *Frontiers in Microbiology* 5.

76. Moore ER, Davie-Martin CL, Giovannoni SJ, & Halsey KH (2020) Pelagibacter metabolism of diatom-derived volatile organic compounds imposes an energetic tax on photosynthetic carbon fixation. *Environmental Microbiology* 22(5):1720-1733.

77. Beiralas R, Ozer N, & Segev E (2023) Abundant Sulfitobacter marine bacteria protect Emiliania huxleyi algae from pathogenic bacteria. *ISME Communications* 3(1).

78. Grover JP (2011) Resource Storage and Competition with Spatial and Temporal Variation in Resource Availability. *The American Naturalist* 178(5):E124-E148.

79. Hutchinson GE (1961) The Paradox of the Plankton. *The American Naturalist* 95(882):137-145.

80. Sommer U (1984) The paradox of the plankton: Fluctuations of phosphorus availability maintain diversity of phytoplankton in flow-through cultures1. *Limnology and Oceanography* 29(3):633-636.

81. Grover JP (1990) Resource Competition in a Variable Environment: Phytoplankton Growing According to Monod's Model. *The American Naturalist* 136(6):771-789.
