# Supplementary material for: Emergent ecology in a microscale model of the surface ocean
Source: mBio. 2024 Oct 9;15(11):e02372-24. doi: 10.1128/mbio.02372-24 (PMC11559031; doi:10.1128/mbio.02372-24)
Supplement: Movie Legends — Legends for supplemental movies. [file mbio.02372-24-s0002.docx]

**Movie Legends**

Movie. S1. Long 2D movie, model illustration. 80 minutes, ×33 speed. See Fig. 1 legend for details and main text for description of events.

Movie. S2. Short 2D movie, model illustration. ~1 minute, real-time speed. See Fig. 1 legend for details and main text for description of events.

Movie. S3. Long 3D movie, model illustration. 80 minutes, ×33 speed. Perspective view, i.e. objects closer to the observer appear bigger, creating a 3D environment. See legend Fig. S2.

Movie. S4. Short 3D movie, model illustration. ~1 minute, real-time speed. Orthographic view, i.e. object’s sizes stay constant regardless of their distance to the observer. See legend Fig. S2.
